# A MYCN-independent mechanism mediating secretome reprogramming and metastasis in *MYCN*-amplified neuroblastoma

**DOI:** 10.1126/sciadv.adg6693

**Published:** 2023-08-23

**Authors:** Hai-Feng Zhang, Alberto Delaidelli, Sumreen Javed, Busra Turgu, Taylor Morrison, Christopher S. Hughes, Xiaqiu Yang, Manideep Pachva, Michael M. Lizardo, Gurdeep Singh, Jennifer Hoffmann, Yue Zhou Huang, Khushbu Patel, Rawan Shraim, Sonia H. Y. Kung, Gregg B. Morin, Samuel Aparicio, Daniel Martinez, John M. Maris, Kristopher R. Bosse, Karla C. Williams, Poul H. Sorensen

**Affiliations:** ^1^ Department of Pathology and Laboratory Medicine, University of British Columbia, Vancouver, BC V6T1Z4, Canada.; ^2^ Department of Molecular Oncology, BC Cancer Agency, Vancouver, BC V5Z1L3, Canada.; ^3^ Faculty of Pharmaceutical Sciences, The University of British Columbia, Vancouver, BC, Canada.; ^4^ Division of Oncology and Center for Childhood Cancer Research, Children's Hospital of Philadelphia, Philadelphia, PA 19104, USA.; ^5^ Department of Pediatrics, Perelman School of Medicine at the University of Pennsylvania, Philadelphia, PA 19104, USA.; ^6^ Department of Biomedical and Health Informatics, Children's Hospital of Philadelphia, Philadelphia, PA 19104, USA.; ^7^ Vancouver Prostate Centre, Vancouver, BC V6H3Z6, Canada.; ^8^ Canada’s Michael Smith Genome Sciences Centre, Vancouver, BC V5Z4S6, Canada.; ^9^ Department of Medical Genetics, University of British Columbia, Vancouver, BC V6T1Z4, Canada.

## Abstract

*MYCN* amplification (*MNA*) is a defining feature of high-risk neuroblastoma (NB) and predicts poor prognosis. However, whether genes within or in close proximity to the *MYCN* amplicon also contribute to *MNA^+^* NB remains poorly understood. Here, we identify that *GREB1*, a transcription factor encoding gene neighboring the *MYCN* locus, is frequently coexpressed with *MYCN* and promotes cell survival in *MNA^+^* NB. GREB1 controls gene expression independently of MYCN, among which we uncover myosin 1B (*MYO1B*) as being highly expressed in *MNA^+^* NB and, using a chick chorioallantoic membrane (CAM) model, as a crucial regulator of invasion and metastasis. Global secretome and proteome profiling further delineates MYO1B in regulating secretome reprogramming in *MNA^+^* NB cells, and the cytokine MIF as an important pro-invasive and pro-metastatic mediator of MYO1B activity. Together, we have identified a putative GREB1-MYO1B-MIF axis as an unconventional mechanism promoting aggressive behavior in *MNA^+^* NB and independently of MYCN.

## Teaser

GREB1, adjacent to the MYCN amplicon, has crucial pro-oncogenic functions independently of MYCN in MYCN-amplified neuroblastoma.

## INTRODUCTION

Neuroblastoma (NB) is the most common pediatric extracranial solid tumor and is responsible for approximately 13% of all pediatric cancer–related deaths (*1*–*3*). These tumors are most commonly found in the adrenal medulla, although they can also arise from the paraspinal sympathetic ganglia of the neck, chest, abdomen, or pelvis (*1*–*3*). High-risk NBs are often associated with widely metastatic disease, leading to a markedly worse prognosis for these tumors (*1*–*3*). *MYCN* amplification occurs in approximately 20% of NB patients and is a poor prognostic factor with associated survival rates of ~30%, in contrast to more than 80% for nonamplified cases (*3*–*5*). As a member of the basic helix-loop-helix (bHLH) family transcription factors, MYCN promotes the expression of proteins involved in cell motility, extracellular matrix degradation, and invasion, thereby facilitating tumor metastasis (*6*). In addition to its canonical function as a transcription factor, *MYCN* transcripts can also mediate oncogenic features as a competing endogenous RNA (ceRNA) for *let-7*, a family of microRNAs with tumor suppressor functions in various human malignancies (*7*).

Although not widely appreciated, it has been documented that genes neighboring the *MYCN* locus, such as *DDX1*, *NBAS*, *GREB1*, *FAM49A*, and *FAM84A*, are frequently coamplified with *MYCN* in *MNA^+^* NB (*8*–*12*). However, whether these genes contribute to *MNA^+^* NB pathogenicity and aggressiveness independently of MYCN or are merely passengers in the oncogenic process remains largely unknown. Among these genes, *GREB1* (growth regulating estrogen receptor binding 1) encodes a transcription factor known to be a crucial estrogen receptor (ER) regulatory factor and oncoprotein in ER*^+^* breast cancer (*13*, *14*). Moreover, GREB1 promotes tumorigenicity in other hormone-dependent cancers, including ovarian cancer, prostate cancer, and endometrial cancer (*15*–*18*), as well as non–hormone-dependent cancers such as hepatoblastoma, the predominant pediatric hepatic neoplasm (*19*). In support of a proto-oncogenic function for GREB1, various fusion genes involving *GREB1* have been identified in uterine tumors resembling ovarian sex-cord tumor (UTROSCT), including *GREB1-NCOA1*, *GREB1-NCOA2*, *GREB1-NR4A3*, *GREB1-SS18*, and *ESR1-GREB1*, although its role in tumorigenesis remains unclear (*20*–*22*).

Myosins are motor proteins that control cell motility through their interaction with actin filaments (*23*). Myosin 1B (MYO1B) is an unconventional myosin that functions by regulating actin assembly in post-Golgi vesicle transport and in endocytic compartments (*24*–*25*). Specifically, it associates with organelles that regulate intracellular trafficking of endosomes, multivesicular bodies, and lysosomes (*24*–*27*). MYO1B has been reported to promote tumorigenesis and metastasis in cancers such as prostate cancer, cervical cancer, glioblastoma, and head and neck squamous cell carcinoma (*27*–*30*). It is up-regulated in metastatic tumor cells, and *MYO1B* knockdown (KD) alters cell morphology and decreases tumor cell invasion (*27*–*30*). However, the underlying molecular mechanisms mediating MYO1B functions in cancer remain poorly understood, nor is it known whether MYO1B plays a role in NB.

Macrophage migration inhibitory factor (MIF) is a pleiotropic cytokine that functions as a hormone, chaperone protein, and enzyme (via an N-terminal proline with tautomerase activity) (*31*–*33*). It is expressed by various cell types, including epithelial, endothelial, and immune cells (*33*). Notably, unlike various cytokines that are secreted upon antigenic stimulation, MIF is constitutively expressed and stored intracellularly, and then undergoes secretion by unknown mechanisms due to lack of an N-terminal leader sequence (*32*, *34*). Secreted MIF binds surface CD74, CD44, and the chemokine receptors CXCR2/4/7, leading to activation of signaling pathways involving RAS–extracellular signal–regulated kinase 1/2 (ERK1/2), SRC, and phosphatidylinositol 3-kinase (PI3K)–AKT (*32*–*33*, *35*). Intracellular MIF is also biologically active and can form a complex with Jab1, a coactivator of AP-1 transcription, thereby inhibiting both Jab1- and stimulus-enhanced AP-1 activity (*36*). The pro-oncogenic role of MIF has been reported for certain types of cancers, such as glioblastoma, melanoma, gastric cancer, and NB (*33*, *35*), enhancing tumor growth, invasiveness, and angiogenesis (*33*, *35*). MIF knockout mice are fertile, and their progeny develop and age normally (*34*), suggesting that MIF blockade in cancer may have limited if any systemic toxicities. However, the mechanism by which MIF is regulated in cancer, particularly its secretion, remains poorly understood.

Here, we uncover that the *GREB1* gene, which lies in close proximity to the *MYCN* amplicon, is frequently coexpressed with *MYCN* in NB. We find that *GREB1* is highly expressed in *MNA^+^* NB at levels comparable to ER*^+^* breast cancer. We define a previously unrecognized GREB1-MYO1B-MIF axis that contributes to the pathobiology of *MNA^+^* NB, but in a manner that is independent of MYCN. Moreover, our integrated secretome and proteome analyses reveal GREB1-induced MYO1B as a major regulator of NB secretome reprogramming, and identify MYO1B-promoted cytokine MIF release as a crucial pro-invasive and pro-metastatic mechanism in *MNA^+^* NB.

## RESULTS

### *GREB1*, a transcription factor–encoding gene neighboring *MYCN*, is highly expressed in *MNA^+^* NB

**Fig. 1.**
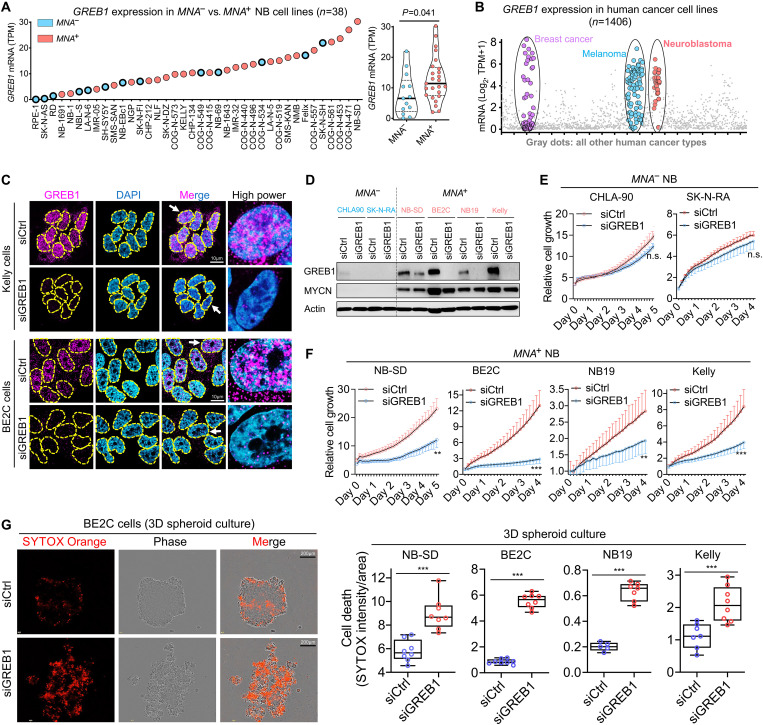
The pro-oncogenic function of GREB1 in *MNA^+^* NB. (**A**) Comparison of *GREB1* expression levels in a panel of *MNA*^−^ and *MNA^+^* NB cell lines. Data were extracted from Gene Expression Omnibus GSE89413. (**B**) The expression profile of *GREB1* in human cancer cell lines plotted based on data from the DepMap database_Expression Public 22Q4. (**C**) Immunofluorescence (IF) analysis of GREB1 in *MNA^+^* NB cells Kelly and BE2C, and cells with *GREB1* gene depletion were used as controls. (**D**) Western blotting analysis of the indicated proteins in NB cells transfected with siCtrl or siGREB1. (**E** and **F**) The impact of *GREB1* gene depletion on cell growth was evaluated by Incucyte in *MNA*^−^ versus *MNA^+^* NB cells (*n* = 6 to 8). (**G**) Anoikis induced by *GREB1* gene depletion was evaluated by Incucyte with SYTOX Orange labeling in 3D spheroid cultures at day 4 (*n* = 5 to 8). For all panels, data are presented as means ± SD. *P* values were determined by two-tailed unpaired Student’s *t* test. n.s., no significance; **P < 0.01; ***P < 0.001.

Genes flanking the *MYCN* locus, such as *DDX1*, *NBAS*, *GREB1*, *FAM49A*, and *FAM84A*, are frequently coamplified with *MYCN* in *MNA^+^* NB (*8*–*12*), but their contributions to *MNA^+^* NB tumorigenesis remain poorly understood. As a first step to address this question, we evaluated the copy number of genes neighboring the *MYCN* locus in a panel of 31 NB lines, of which 21 are *MNA^+^*, as well as a cohort of 554 NB patient samples that contains 255 *MNA^+^* tumors. This confirmed coamplification of *MYCN* with *DDX1*, *NBAS*, *FAM49A*, *GREB1*, and *FAM84A* at variable levels of coamplification (fig. S1, A and B), in agreement with previous studies (*8*–*12*). Among these, *GREB1* was of particular interest as it is the only transcription factor–encoding gene among this group. *GREB1* was most strongly associated with *MYCN* expression (*r* = 0.537 and *P* = 0.002; fig. S1C) and is up-regulated in *MNA^+^* NB compared with *MNA*^−^ NB (Fig. 1A), although it was not the most frequently coamplified gene with *MYCN* in our analyses. Nonetheless, we observed a highly significant correlation between *GREB1* and *MYCN* expression in five independent cohorts of NB tumor samples (fig. S1D). Notably, *GREB1* expression is not induced by MYCN, as *MYCN* depletion in Tet21N cells that express *MYCN* cDNA under the control of a Tet-Off system failed to reduce *GREB1* levels and instead showed a trend toward increased *GREB1* expression (fig. S1E). Moreover, among a large panel of human cancer cell lines (*n* = 1406), NB cells are among the highest *GREB1* expressors (Fig. 1B), comparable to breast cancer and melanoma that are known to highly express GREB1 (*13*–*14*, *17*). Finally, compared with normal adrenal glands where NB most commonly originates, *GREB1* expression is significantly increased across multiple cohorts of NB tumors (fig. S1F). Collectively, these results show that *GREB1* is highly expressed in NB, particularly in *MNA^+^* cases, pointing to a previously uncharacterized role for this gene in NB.

Next, we confirmed the nuclear localization of GREB1 in *MNA^+^* NB cells (Fig. 1C), although some cytoplasmic staining was also observed (Fig. 1C), in support of its recently uncovered cytoplasmic function in ER*^+^* breast cancer as an O-GlcNAc glycosyltransferase (*14*). Functionally, while *GREB1* KD had minimal impact on *MNA*^−^ NB cells including CHLA-90 and SK-N-RA (Fig. 1, D and E, and fig. S2A), it significantly inhibited cell growth in multiple *MNA^+^* NB cell lines, including NB-SD, BE2C, NB19, and Kelly (Fig. 1, D and F), which was accompanied by marked cell death (fig. S2B). Nevertheless, despite significantly higher *GREB1* expression in *MNA^+^* NB compared with *MNA*^−^ NB, not all *MNA*^−^ cells are GREB1-low (Fig. 1A), including NB69, as we verified (fig. S2G, left panel). However, *GREB1* KD failed to inhibit cell growth or enhance death in NB69 cells (fig. S2G, right panels). This suggests that these phenotypes are not controlled by GREB1 in NB69 cells, distinct from its functions in *MNA^+^* NB cells. Notably, rather than harboring a *MYCN* amplification, NB69 is uniquely characterized by *c-MYC* overexpression (OE) (fig. S2H), as recently reported (*37*). However, c-MYC did not promote *GREB1* expression in NB69 cells, and unexpectedly, *c-MYC* depletion actually significantly increased *GREB1* levels (fig. S2I). This is consistent with an inverse correlation between *c-MYC* and *GREB1* in NB cell lines (fig. S2J) as well in a large panel of cancer cell lines (fig. S2K), in contrast to the positive correlation observed between *MYCN* and *GREB1* in NB (fig. S2, J and K).

Given that *anoikis* resistance is an essential feature of aggressive tumor cells and is crucial for metastasis (*38*–*40*), we also evaluated whether GREB1 controls *anoikis* in three-dimensional (3D) cultures. Strikingly, *GREB1* KD led to marked *anoikis* and disruption of spheroid formation in each of the above *MNA^+^* NB cell lines (Fig. 1G), and the effects on cell death in 3D cultures were more pronounced than in 2D cultures (fig. S2C). Finally, using the Tet21N NB line expressing the *MYCN*–Tet-Off system (*41*), we found that MYCN did not govern the expression (fig. S2D) or biological functions of GREB1 (fig. S2, E and F), arguing that MYCN acts independently of GREB1. Together, these results demonstrate a previously unknown role for GREB1 in the growth and survival of *MNA^+^* NB cells. Moreover, the sensitivity to *GREB1* KD was closely associated with higher GREB1 expression in the *MNA^+^* NB cells compared with the *MNA*^−^ NB cell lines tested (Fig. 1D), and enforced *MYCN* expression did not confer GREB1 dependency (fig. S2, D to F).

### GREB1 activates a MYCN-independent gene signature in *MNA^+^* NB

**Fig. 2.**
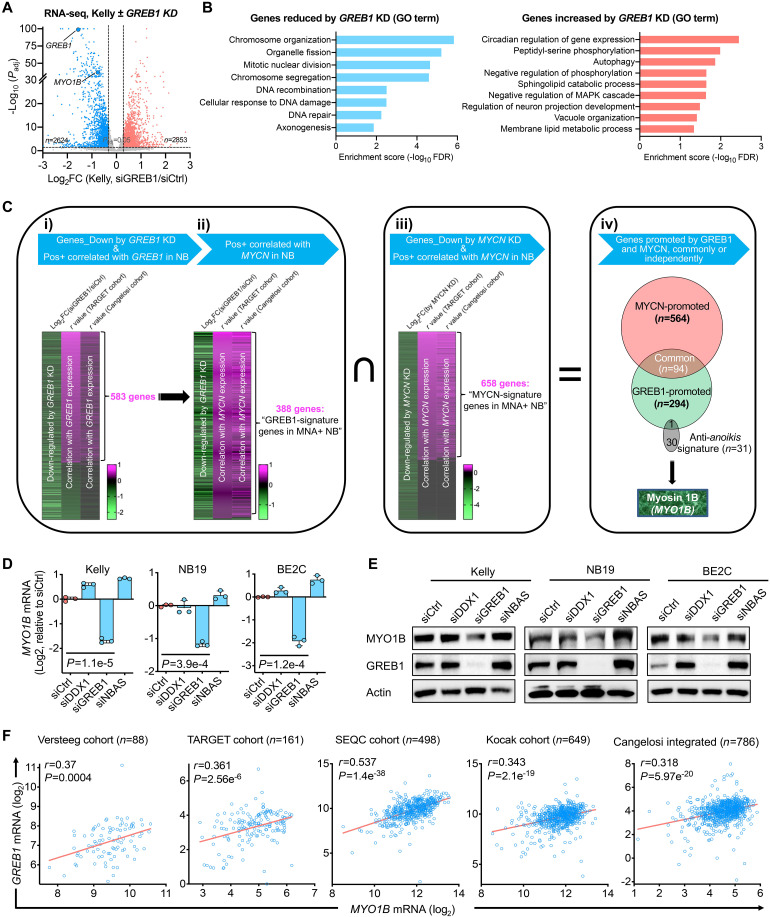
Identification of a GREB1-controlled gene signature in *MNA^+^* NB independent of MYCN. (**A**) RNA-seq analysis in Kelly cells ± *GREB1* knockdown (KD). The analysis was performed in triplicate. (**B**) Gene Ontology (GO) analysis of the gene sets significantly reduced/increased by *GREB1* KD in Kelly cells [genes with log_2_ fold change (FC) of >0.3 or <−0.3 were included]. GO term analysis was performed using the PANTHER database, and the GO-Slim Biological Process was analyzed. (**C**) Gene sets and procedures used for the integrated analysis, which identified a GREB1-controlled gene signature in *MNA^+^* NB independent of MYCN. (**D** and **E**) MYO1B expression changes upon KD of the indicated genes assessed by quantitative polymerase chain reaction (D) and immunoblotting (E). (**F**) Correlation between *GREB1* and *MYO1B* mRNA expression in five cohorts of NB patient samples based on data derived from the R2 database. Data presented are means ± SD; *P* values were determined by two-tailed unpaired Student’s *t* test. Pearson coefficient analysis was performed to determine correlations between two variables.

The above observations prompted us to hypothesize that, in addition to MYCN, coexpression of GREB1 may transcriptionally regulate a distinct set of genes that also contribute to *MNA^+^* NB. To identify such a gene set, we performed RNA sequencing (RNA-seq) in *MNA^+^* cells, namely, Kelly cells ± *GREB1* KD, to evaluate transcriptomic alterations. This uncovered 2624 and 2853 genes significantly reduced or increased, respectively, by *GREB1* KD (Fig. 2A and table S1). Gene Ontology (GO) term analysis revealed potential GREB1 regulation of biological processes such as chromosome organization, DNA damage repair, and axonogenesis (Fig. 2B). The top enriched GO terms among genes suppressed by GREB1 included circadian regulation of gene expression, the dysregulation of which affects various hallmarks of cancer (*42*–*43*), as well as serine phosphorylation and autophagy (Fig. 2B). These previously unknown findings warrant future in-depth studies.

Next, we wished to down-select genes from the RNA-seq analysis that might be crucial for mediating the pathobiological functions of GREB1 in NB. First, we integrated genes from RNA-seq that were significantly altered by *GREB1* KD with those genes that are significantly correlated with *GREB1* expression across two large NB patient cohorts, namely, the TARGET cohort (*n* = 161; table S2) and Cangelosi cohort (*n* = 768; table S3), and plotted the data as heatmaps (Fig. 2Ci and fig. S3A). This approach effectively distinguished between GREB1-induced and GREB1-suppressed genes; for example, the gene set down-regulated by *GREB1* KD showed a higher positive correlation with *GREB1* in both NB cohorts, compared with genes up-regulated by *GREB1* KD (fig. S3B). To identify genes that promote GREB1-mediated functions, we focused on the 583 genes from this integrated analysis for further studies (Fig. 2Ci and table S4). Second, to uncover genes that are potential drivers in *MNA^+^* NB, we then integrated the above 583 gene set with genes positively correlated with *MYCN* in both cohorts of NB patients (Fig. 2Cii and tables S5 and S6). Strikingly, a large percentage, i.e., 388 of the 583 GREB1-promoted genes (66.6%), were positively correlated with *MYCN* in both NB cohorts (Fig. 2Cii), echoing our finding that *GREB1* and *MYCN* are frequently coexpressed (fig. S1D). These 388 genes are hereby denoted as “GREB1-signature genes in *MNA^+^* NB” (table S7).

Third, we leveraged publicly available transcriptome data on inducible *MYCN* depletion, i.e., RNA-seq of Tet21N NB cells ± *MYCN* KD, which identified 2867 reduced and 624 increased genes (*41*) (fig. S3, C and D, and table S8). We then integrated these 2867 MYCN-promoted genes with genes that are positively correlated with *MYCN* expression across the same two large cohorts of NB patients, uncovering 658 genes, denoted herein as “MYCN-signature genes in *MNA^+^* NB” (Fig. 2Ciii and table S9). Finally, to uncover gene sets potentially co-regulated by GREB1 and MYCN, or controlled independently by each in *MNA^+^* NB, we integrated the above 388 GREB1-signature genes and the 658 MYCN-signature genes (Fig. 2Civ). We found 94 common genes potentially co-regulated by both MYCN and GREB1, whereas the vast majority of those gene gets are controlled independently by GREB1 (i.e., 294 of 388, 75.8%) or MYCN (i.e., 564 of 658, 85.7%) (Fig. 2Civ).

### MYO1B is controlled by GREB1 in MNA*^+^* NB in a MYCN-independent manner

Given the crucial role of *anoikis* suppression in metastasis (a major driver of patient mortality) (*38*–*40*), plus our observation that GREB1 suppresses *anoikis* (Fig. 1G), we sought to identify key *anoikis* suppressors among the above signature genes. Thus, we integrated the GREB1- and MYCN-signature gene sets with a 31-protein “*anoikis* suppressor signature” that we recently reported (*40*) (Fig. 2Civ). Strikingly, we identified *MYO1B* (encoding myosin 1B) as the only gene in the *anoikis* signature, which was present in the set of “GREB1-signature genes in *MNA^+^* NB” but not in the set of “MYCN-signature genes in *MNA^+^* NB” (Fig. 2Civ).

To directly test if GREB1 regulates *MYO1B* expression in *MNA^+^* NB, we depleted *GREB1* (as well as *DDX1* and *NBAS* as *MYCN* amplicon-associated controls) in three *MNA^+^* NB cell lines (gene depletion is shown in fig. S4A) and assessed MYO1B expression. KD of *GREB1* but neither *DDX1* nor *NBAS* reduced *MYO1B* mRNA (Fig. 2D) as well as protein levels (Fig. 2E). We found direct binding of GREB1 to the *MYO1B* locus at exon 3, as evidenced by both publicly available GREB1 chromatin immunoprecipitation sequencing (ChIP-seq) data in MCF7 breast cancer cells (fig. S4B) and our GREB1 CUT&RUN analysis [i.e., Cleavage Under Targets and Release Using Nuclease, an alternative approach for in situ ChIP analysis (*44*)] in *MNA^+^* Kelly cells (fig. S4, C and D). Moreover, we validated the GREB1-MYO1B link using a publicly available dataset of LNCaP prostate cancer cells ± *GREB1* KD (*18*), demonstrating a significant reduction in *MYO1B* expression upon *GREB1* KD (fig. S4E). Accordingly, we observed a highly significant correlation between *GREB1* and *MYO1B* mRNA expression across five different NB tumor cohorts (Fig. 2F), as well as in multiple prostate adenocarcinoma cohorts in public datasets (fig. S4F). Together, these findings strongly point to *MYO1B* as a transcriptional target of GREB1 in NB and other cancers.

To rule out a role for MYCN in regulating MYO1B in *MNA^+^* NB, we performed shRNA or siRNA KD of *MYCN*, neither of which decreased MYO1B protein expression in the *MNA^+^* NB cell lines NGP, Kelly, and BE2C (fig. S5A). Furthermore, using Tet21N cells, doxycycline-induced *MYCN* depletion failed to reduce MYO1B expression (fig. S5B). Finally, MYCN OE in *MNA*^−^ CHLA90 cells did not induce MYO1B expression (fig. S5C). Together, these data indicate that GREB1, but not MYCN, transcriptionally regulates *MYO1B* in *MNA^+^* NB cells.

### MYO1B is highly expressed in MNA*^+^* NB and is associated with poor clinical outcome

**Fig. 3.**
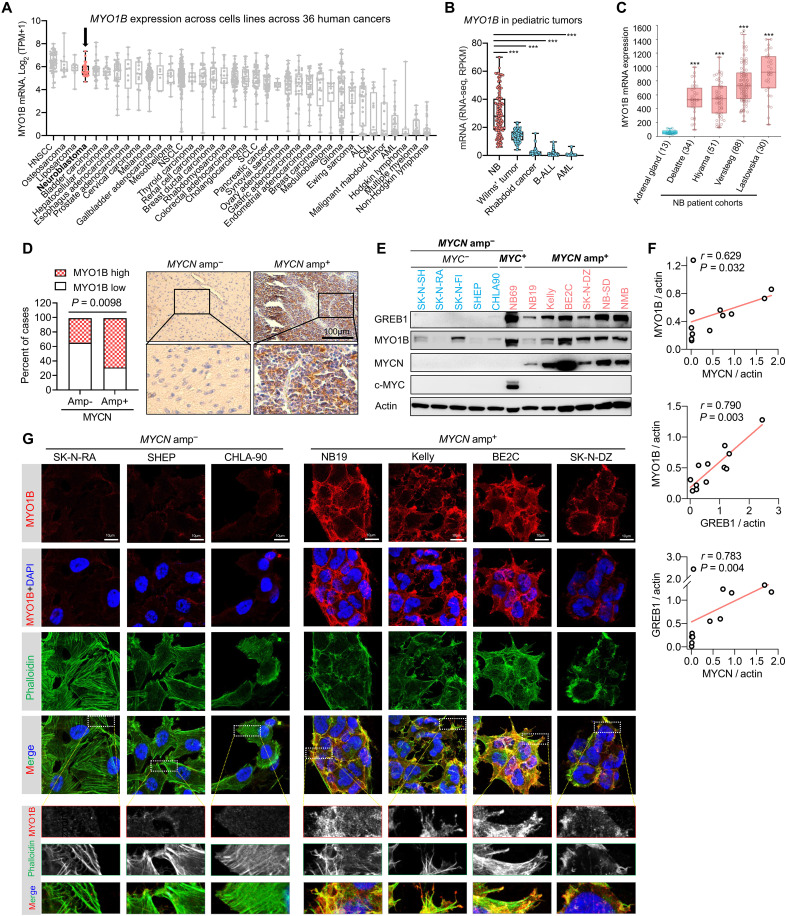
MYO1B is strongly expressed in NB with *MYCN* amplification. (**A**) Expression profile of *MYO1B* in human cancer cell lines plotted based on data from the DepMap database_Expression Public 21Q4. (**B**) The expression profile of *MYO1B* in pediatric cancers in the TARGET project cohort was extracted from cBioPortal database. (**C**) Comparison of *MYO1B* mRNA levels in four cohorts of NB patient samples and nontumor adrenal gland samples. Plots were derived from the R2 database. (**D**) Statistics (left panel) and representative images (right panel) showing MYO1B expression evaluated by immunohistochemistry (IHC) in *MNA*^−^ and *MNA^+^* NB. (**E** and **F**) Protein expression of the indicated markers in a panel of NB cell lines was assessed by immunoblotting, and correlation among the markers normalized to an actin loading control was analyzed (F). (**G**) Confocal microscopic analysis of the expression level of MYO1B and its colocalization with actin cytoskeleton as assessed by TRITC-phalloidin staining. Differences between groups were determined by two-tailed unpaired Student’s *t* test. ***P < 0.001. Fisher’s exact test was used to determine the associations between two categorical variables in two groups. Pearson coefficient analysis was performed to determine correlations between two variables.

Among various human cancers, NB is among the highest MYO1B expressors (Fig. 3, A and B), and *MYO1B* expression is sharply increased in multiple cohorts of NB tumors compared with normal adrenal glands (Fig. 3C). Next, we verified that high expression of MYO1B is a feature of *MNA^+^* NB. First, we observed consistently higher *MYO1B* mRNA expression in *MNA^+^* compared to *MNA*^−^ tumors, along with positive correlations across five NB cohorts, although statistical significance was not reached in all cohorts (fig. S5, D and E). While we observed a relatively moderate correlation between *MYO1B* and *MYCN*, this is likely a secondary effect, reflecting the strong correlation between *GREB1* and *MYCN* in *MNA^+^* NB. Second, we evaluated MYO1B protein levels by immunohistochemistry (IHC) in 137 NB specimens with known *MNA* status, revealing a significant correlation between *MNA* and strong MYO1B staining (*P* = 0.0098; Fig. 3D). Specifically, *MNA^+^* NB tumors were typically MYO1B-high (H-score > 20), while *MNA*^−^ tumors tended to be MYO1B-low (Fig. 3D). Third, MYO1B immunoblotting in a panel of 12 NB cell lines with or without *MNA* again revealed a strong correlation among MYO1B, MYCN, and GREB1 (Fig. 3, E and F). Moreover, immunofluorescence (IF) revealed intense MYO1B staining in *MNA^+^* cells but only minimal staining in *MNA*^−^ cells (Fig. 3G). Finally, we observed strong colocalization between MYO1B and actin structures in *MNA^+^* cells, in particular at cell protrusions (see boxed inserts in Fig. 3G), supporting the reported role of MYO1B in regulating actin assembly and dynamics (*27*, *45*–*48*).

**Fig. 4.**
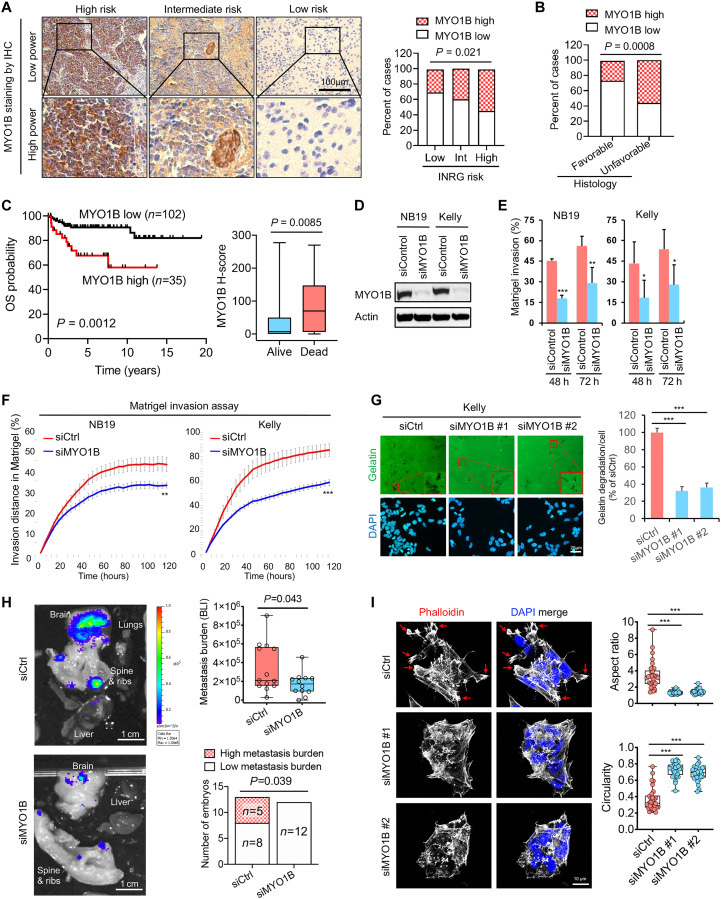
MYO1B correlates with poor prognosis and promotes the invasive and metastatic capacity of *MNA^+^* NB. (**A**) Representative images (left panel) and statistics (right panel) showing MYO1B expression evaluated by IHC in NB with different INRG risks. (**B**) Statistics showing MYO1B expression assessed by IHC in NB with favorable versus unfavorable histology. (**C**) Prognostic significance of MYO1B protein expression (by IHC staining) in a cohort of 137 NB patients. (**D** to **F**) Impact of *MYO1B* depletion (D) on cell invasion through Matrigel was evaluated using ibidi four-well culture inserts (E) and Essen BioScience Incucyte 96-well scratch wound invasion assay (F) (*n* = 5 to 8). (**G**) Impact of *MYO1B* depletion on gelatin degradation capacity (*n* = 5). (**H**) Left panel: The impact of *MYO1B* depletion on the metastatic capacity of NB cells (luciferase-expressing Kelly cells) was evaluated using the chick embryo chorioallantoic membrane (CAM) metastasis model. Right upper panel: Metastasis burden was measured by bioluminescent intensity (BLI) in each embryo; right lower panel: BLI over 5 × 10^5^ was regarded as high metastasis burden. (**I**) The impact of *MYO1B* depletion on NB cell (Kelly) morphology was determined by actin cytoskeleton staining using phalloidin, and the aspect ratio and circularity were assessed using ImageJ software. Differences between groups were determined by two-tailed unpaired Student’s *t* test. *P < 0.05, **P < 0.01, ***P < 0.001. Fisher’s exact test was used to determine the associations between two categorical variables in two groups. Log-rank test was used in Kaplan-Meier survival analysis.

We next explored whether MYO1B is linked to NB progression. First, in the above cohort of 137 NB specimens, high MYO1B protein expression (i.e., H-scores > 20) as assessed by IHC is associated with increased INRG (International Neuroblastoma Risk Group) risk (*P* = 0.021; Fig. 4A) and unfavorable tumor histology (*P* = 0.008; Fig. 4B). Second, high MYO1B protein expression correlates significantly with poor prognosis (*P* = 0.0012; Fig. 4C, left panel), and NB tumors in deceased patients had significantly higher MYO1B levels (*P* = 0.0085; Fig. 4C, right panel). Together, these findings strongly support a role for MYO1B in NB tumorigenesis and suggest a role in aggressive behavior. Of further note, we found that among 33 human cancer types in a publicly available GEPIA database (http://gepia.cancer-pku.cn), *MYO1B* is significantly up-regulated across 10 human cancers compared to their respective normal control tissues, with fold changes ranging from 2.1 to 37.6 (fig. S6A). These findings suggest a potentially broader role for MYO1B in oncogenesis.

### MYO1B promotes NB cell invasiveness and metastatic capacity

We next asked how MYO1B might be promoting NB oncogenesis. Unlike its upstream regulator GREB1, MYO1B inactivation had minimal or no impact on cell proliferation (fig. S6B) or cell death (fig. S6C). In contrast, *MYO1B* depletion in the same *MNA^+^* NB cells strongly inhibited cell invasiveness (Fig. 4, D to F) and markedly reduced gelatin degradation capacity by NB cells (Fig. 4G), suggesting that MYO1B might confer metastatic capacity. To more directly probe this possibility in vivo, we used a chick embryo model previously used to evaluate the metastatic capacity of NB and other cancers (*49*–*50*). Luciferase-expressing Kelly cells with or without *MYO1B* depletion were intravenously injected into chick chorioallantoic membranes (CAM), and metastatic burden was measured by In Vivo Imaging System (IVIS)–based bioluminescent detection. *MYO1B* depletion significantly reduced the metastatic spread of NB cells, as reflected by reduced metastatic burden in each embryo (Fig. 4H, upper-right panel) and reduction of embryos with high metastatic burden (Fig. 4H, lower-right panel). Loss of metastatic capacity was confirmed by hematoxylin and eosin staining and IHC for the NB marker, NCAM, in serial sections (fig. S6D). Moreover, *MYO1B* depletion was also associated with a decrease in NB cell extravasation, but the difference did not quite reach statistical significance (fig. S6E).

We found that the pro-invasive function of MYO1B is not restricted to *MNA^+^* NB cells. Specifically, MYO1B OE in *MNA*^−^ SHEP NB cells also significantly enhanced cell invasiveness in vitro (fig. S7, A and B) and sharply increased cell extravasation in the CAM model (fig. S7C), accompanied by a strong trend toward increased bone metastasis (fig. S7D), although no significant changes were observed in overall metastasis burden. Notably, we experienced difficulties in obtaining metastatic signals with the *MNA*^−^ cell line compared with Kelly (*MNA^+^*) cells, and the relatively weak basal colonization capacity by *MNA*^−^ cells upon extravasation may explain the less robust changes in metastasis observed in this *MNA*^−^ NB model. Moreover, in a distinct non-NB cell model, i.e., murine NIH3T3 fibroblasts transformed by the oncogenic ETV6-NTRK3 (EN) tyrosine kinase that are also MYO1B positive (fig. S7E), *Myo1b* depletion by distinct small interfering RNAs (siRNAs) or short hairpin RNAs (shRNAs) each significantly diminished invasiveness (fig. S7F).

Finally, *MYO1B* depletion in *MNA^+^* Kelly cells induced marked changes in cell morphology and cytoskeletal structures (Fig. 4I), consistent with reduced invasive and metastatic features (*51*–*52*). Specifically, MYO1B inactivation blunted the formation of large spike-like protrusions and highly polymerized cortical actin filaments (Fig. 4I, left panel; see arrows). This was accompanied by marked decreases in the aspect ratio (i.e., the ratio of maximum diameter/minimum diameter) and an increase in cell circularity (Fig. 4I, right panel). Together, these data strongly support the notion that MYO1B promotes features of tumor cell invasiveness and metastatic capacity in NB cells.

### MYO1B is an important regulator of the secretome in NB

**Fig. 5.**
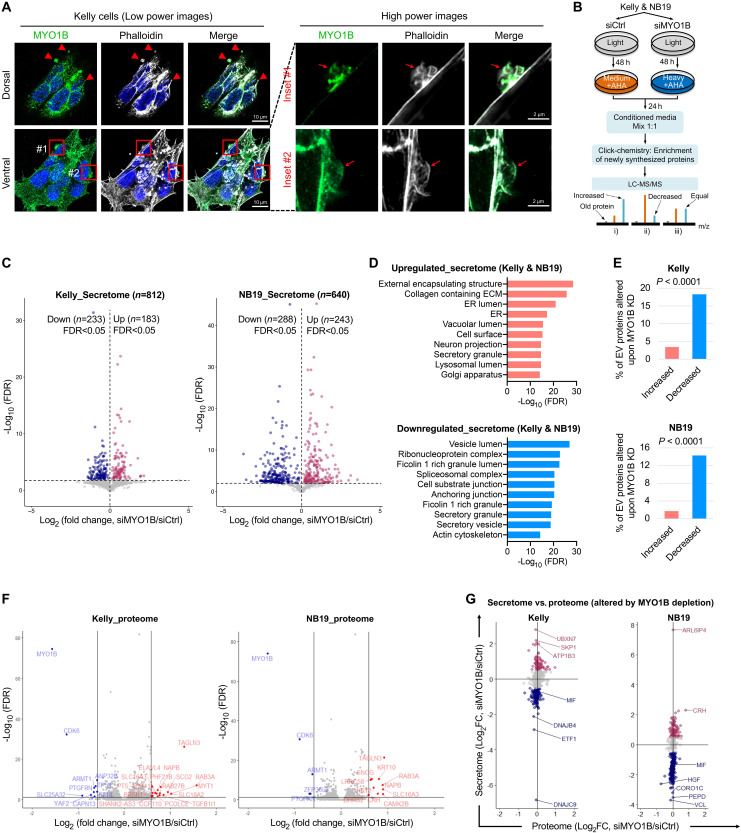
Identification of MYO1B, an important regulator of secretome reprogramming in NB. (**A**) Colocalization of MYO1B with cytoskeletal structures on NB cell surface was evaluated by confocal microscopy. (**B**) Schematic showing the procedures for global secretome evaluation by pSILAC-Click (i.e., pulsed stable isotope labeling with amino acids in cell culture, or pSILAC, combined with Click chemistry) and mass spectrometry (MS). (**C**) Global secretome changes upon *MYO1B* depletion determined by pSILAC-Click described in the procedures in (B) (*n* = 3 independent replicates). (**D**) Gene set enrichment analysis on proteins commonly up-regulated or down-regulated by *MYO1B* depletion in the secretome of NB19 and Kelly cells. (**E**) Percentage of extracellular vesicle (EV)–associated proteins present in the NB secretome that were found increased or decreased by *MYO1B* depletion. (**F**) Global proteome changes upon *MYO1B* depletion were determined by mass spectrometry in both Kelly and NB19 cells (*n* = 3 independent replicates). (**G**) Comparison of global secretome and proteome changes affected by *MYO1B* depletion in NB cells. Note that the scales on *x* and *y* axes in each plot are identical. Fisher’s exact test was used to determine the associations between two categorical variables in two groups.

Unexpectedly, we noticed an enrichment of MYO1B in structures with the appearance of budding vesicles on the cell surfaces of NB cells by confocal IF (Fig. 5A), which we also observed in EN- and KRas^G12V^-transformed NIH3T3 cells (fig. S8, A and B), pointing to a possible role of MYO1B in cell secretory activity. However, a function for MYO1B in secretome reprogramming has not been previously reported in cancer cells. To further test this possibility, we used pSILAC-Click (i.e., pulsed stable isotope labeling with amino acids in cell culture, or pSILAC, combined with Click chemistry) to specifically label and purify cell-derived proteins in conditioned medium (CM) (Fig. 5B). This approach enables analysis of the acute secretome in cells cultured under normal growth medium instead of serum starvation conditions that are required by more conventional approaches, overcoming interference by large amounts of albumin proteins in the CM (*53*–*54*). We identified 812 and 640 proteins in the secretomes of Kelly and NB19 NB cells, respectively (tables S10 and S11). *MYO1B* depletion led to marked secretome alterations, with 51.2% of proteins (233 up and 183 down) and 83.0% (288 up and 243 down) significantly changed in Kelly and NB19 cells, respectively [false discovery rate (FDR) < 0.05; Fig. 5C]. Moreover, concordant secretome changes were observed across both cell lines (*r* = 0.817, *P* < 0.0001; fig. S9A). Gene set enrichment analysis (GSEA) of the latter revealed commonly up-regulated secreted proteins that were highly enriched in components of the external encapsulating structure, collagen-containing ECM (extracellular matrix), and ER proteins (FDR < 6 × 10^−18^; Fig. 5D, upper panel). Notably, and consistent with the presence of MYO1B in budding vesicles (Fig. 5A and fig. S8, A and B), secretome proteins that were down-regulated by *MYO1B* depletion included components of vesicle lumens, Ficolin 1–rich granule lumens, as well as secretory granules and secretory vesicles (FDR < 2 × 10^−19^; Fig. 5D, lower panel). Moreover, there was preferential down-regulation of extracellular vesicle protein contents upon *MYO1B* depletion in NB cells (*P* < 0.0001; Fig. 5E).

Next, we checked if secretome changes following *MYO1B* depletion were due to reduced expression of secreted proteins in NB cells. To this end, we performed tandem mass tag (TMT)–based mass spectrometry (MS) to identify global proteomic differences in NB19 and Kelly cells with or without *MYO1B* KD (tables S12 and S13), which showed concordant proteomic changes across both cell lines (*r* = 0.591, *P* < 0.0001; fig. S9B). However, MYO1B loss induced only a very limited number of overall proteomic changes, with <10 proteins reduced and <20 proteins increased >1.5-fold in both cell lines, and these did not include changes in secreted proteins (Fig. 5F). There was a markedly broader distribution of proteins on the *y* axis representing changes in secreted proteins, as compared with corresponding proteome alterations as represented on the *x* axis (Fig. 5G). This suggests that rather than affecting the actual synthesis of secreted proteins, MYO1B regulation of the secretome in NB cells occurs through a different and as yet unknown mechanism.

### Secretion of the MIF cytokine is regulated by MYO1B and stimulates NB cell invasion and metastasis

Among proteins commonly and significantly reduced in the secretomes of both NB19 and Kelly cells following *MYO1B* depletion, GSEA identified seven proteins that belonged to the cytokine and growth factor category, including MIF, DKK1, SST, SLIT2, HGF, GPI, and SEMA3A (table S14). Similar to *MYO1B*, *MIF* is also highly expressed in *MNA^+^* NB (fig. S10, A and B) and is reported to promote NB tumor progression (*33*). However, in contrast to *MYO1B* that is not controlled by MYCN (fig. S5, A to C), ChIP-seq of a panel of *MNA^+^* NB cells revealed strong binding of MYCN (but not GREB1) across the *MIF* locus, and two E-box elements (CANNTG) were identified in exons 1 and 2, respectively (fig. S10C). Moreover, *MYCN* depletion significantly reduced *MIF* expression in a dose-dependent manner (fig. S10D), and high *MIF* expression is associated with poor prognosis in NB patients in multiple cohorts (fig. S10E). We therefore next asked to what extent MIF secretion mediates the biological functions of MYO1B in *MNA^+^* NB.

**Fig. 6.**
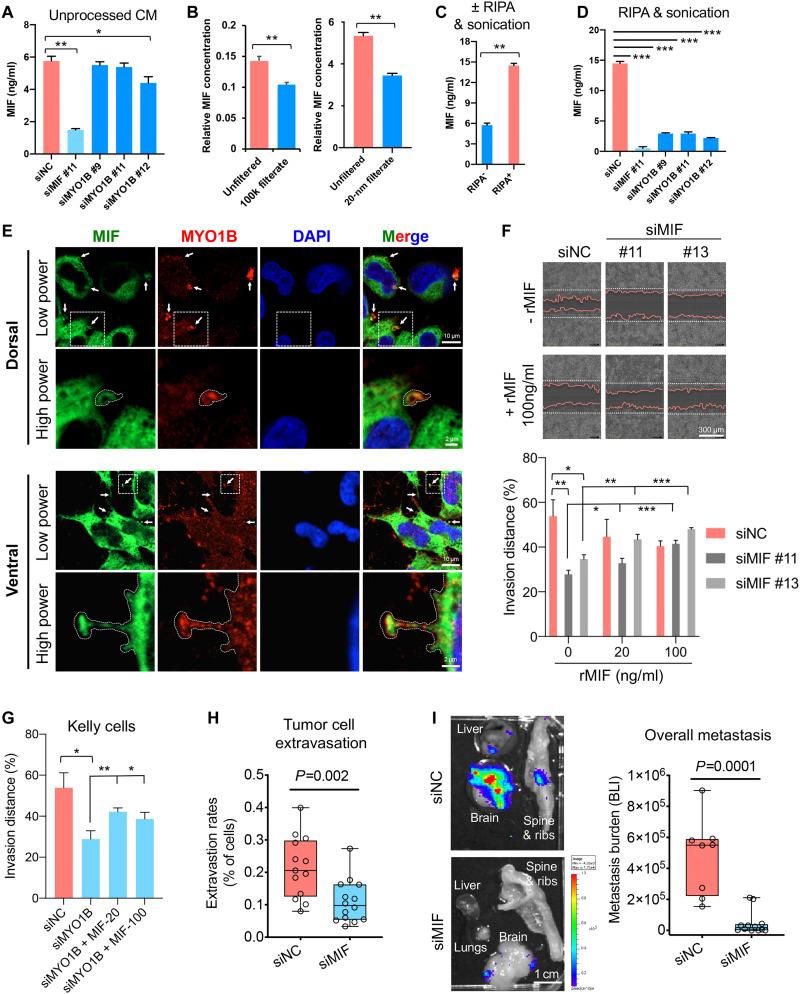
MIF secretion enhanced by MYO1B promotes NB cell invasion and metastasis. (**A**) Evaluation of MIF concentrations by ELISA in unprocessed conditioned medium (CM) from the indicated siRNA-transfected Kelly cells (*n* = 4). (**B** and **C**) Impact of filtration by filters with 100 kDa or 20-nm pore sizes (B) or RIPA + sonication treatment (C) on MIF concentrations in CM samples from Kelly cells (*n* = 4). (**D**) Impact of *MIF* or *MYO1B* depletion on MIF concentrations in RIPA + sonication–treated CM samples from Kelly cells (*n* = 3). (**E**) Colocalization of MIF with MYO1B in Kelly cells was evaluated by IF confocal microscopy. (**F** and **G**) Impact of *MIF* or *MYO1B* depletion ± recombinant MIF (rMIF) treatment on cell invasion through Matrigel was evaluated by Incucyte (*n* = 5 to 8). (**H** and **I**) Impact of *MIF* depletion on the extravasation (H) and metastatic capacity (I) of luciferase-expressing Kelly cells was evaluated using the chick embryo CAM metastasis model. The metastasis burden was measured by BLI. Differences between groups were determined by two-tailed unpaired Student’s *t* test. *P < 0.05, **P < 0.01, ***P < 0.001.

We first tested whether MYO1B controls MIF secretion using ELISA (enzyme-linked immunosorbent assay). However, *MYO1B* depletion using independent siRNAs led to only minimal decreases in MIF secretion when CM was directly used for ELISA after cell debris elimination (Fig. 6A). Since our original secretome profiling and identification of MIF was performed using CM samples that were processed in urea lysis buffer as per our pSILAC-Click protocol, we reasoned that the lysis procedure might be critical for detecting MIF. For example, if MIF is encapsulated in secreted vesicles, it might be undetectable by ELISA, even if regulated by MYO1B. We therefore used filters with 100 kDa or 20-nm pore sizes, which are much larger than the molecular size of MIF [12 kDa or ~3 nm in diameter (*55*), which trapped a large proportion of inputted MIF in the CM] (Fig. 6B). Therefore, we lysed the CM samples with a harsh detergent radioimmunoprecipitation assay (RIPA) buffer followed by sonication before ELISA analysis, which revealed a marked increase in the yield of MIF by ELISA (Fig. 6C). Using this method, we observed a marked reduction of MIF secretion in NB cells after *MYO1B* depletion (Fig. 6D). Moreover, MYO1B and MIF colocalized in budding vesicles on both dorsal and ventral sections of NB cell cultures (Fig. 6E), pointing to the possibility that MYO1B is directly involved in MIF secretion.

We next validated whether MIF mediates, at least partially, the pro-invasive function of MYO1B. First, as observed with *MYO1B* KD, *MIF* depletion had minimal effects on cell growth (fig. S10, F and G), but significantly reduced NB cell invasiveness in Matrigel, which could be rescued by recombinant MIF (rMIF) in a concentration-dependent manner (Fig. 6F). Moreover, rMIF significantly restored cell invasiveness to *MYO1B*-depleted cells (Fig. 6G). Using the CAM metastasis model, we confirmed an important role for MIF in NB metastasis in vivo. *MIF* depletion in NB cells markedly inhibited tumor cell extravasation (*P* = 0.002; Fig. 6H), as well as metastatic burden in embryos (*P* = 0.0001; Fig. 6I and fig. S11A). Together, these data point to a model whereby MYO1B increases secretion of MIF (and likely other factors) to increase *MNA^+^* NB cell pro-invasive activity. Moreover, MYCN appears to contribute to a putative GREB1-MYO1B-MIF axis, at least in part, through transcriptional induction of MIF, which is then secreted by NB cells in a MYO1B-dependent manner, to control NB invasiveness and potential aggressive behavior. Unfortunately, high rates of cell death and lysis of NB cells with *GREB1* KD (Fig. 1G and fig. S2B) precluded a reliable assessment of the role of GREB1 in this process.

### *GREB1* and *MYO1B* are overexpressed in *MNA^+^* medulloblastoma

To further validate our finding that *MNA^+^* cancers may deploy a GREB1-dependent mechanism to drive expression of pro-oncogenic genes such as *MYO1B*, independently of MYCN, we investigated medulloblastoma (MB), a childhood brain tumor that also harbors recurrent *MNA* (*56*–*58*). Among the four major MB subgroups (Wnt, Shh, Group 3, and Group 4), *MYCN* is most frequently amplified in the Shh subgroup (*56*–*58*). Accordingly, in two large independent MB cohorts, we observed significantly higher *MYCN* expression in the Shh subgroup compared to other MB subgroups, in particular Groups 3 and 4 (fig. S12, A and B, left panel). In keeping with our hypothesis, the Shh subgroup had significantly higher levels of *GREB1* as well as *MYO1B* compared to Group 3 and 4 MBs (fig. S12, A and B, middle and right panels). Of note, the Wnt subgroup had the highest expression of *MYO1B* in these MB subgroups (fig. S12, A and B), suggesting a potential role for the Wnt signaling pathway in addition to GREB1, which warrants further investigation. Nonetheless, we found significant correlations among *MYCN* and *GREB1*, *MYCN* and *MYO1B*, as well as *GREB1* and *MYO1B* in these MB cohorts (fig. S12, C and D). Together, these findings further support the notion that GREB1 mediates the pathobiological consequences of *MNA* in at least some distinct cancers, including both NB and MB.

## DISCUSSION

NB patients with *MYCN* amplification (*MNA^+^*) are well documented to have dismal survival rates (*3*–*5*). Extensive literature indicates that MYCN promotes NB cell aggressiveness by maintaining embryonic features such as extended self-renewal, augmented apoptotic resistance and metabolic plasticity, and enhanced metastatic capacity (*6*). Notably, genes neighboring the *MYCN* locus are frequently coamplified with *MYCN* in *MNA^+^* NB (*8*–*12*). Whether and how these genes contribute to the pathobiology of *MNA^+^* NB independently of MYCN has remained elusive. Here, we report that the transcription factor–encoding gene *GREB1* neighboring *MYCN* on chromosome 2p24.3 is frequently coexpressed with *MYCN* and functionally important for *MNA^+^* NB. Further, we uncovered a previously unknown GREB1-MYO1B axis that is strongly up-regulated in *MNA^+^* NB, and that MYO1B enhances the invasive and metastatic capacity of *MNA^+^* NB cells. Finally, we delineated that MYO1B plays a crucial role in secretome reprogramming in *MNA^+^* NB, and identified that secretion of the cytokine MIF is promoted by MYO1B as an important metastatic driver in *MNA^+^* NB.

The *GREB1* gene is highly responsive to sex hormones, including estrogen, progesterone, and androgen, and its promoter harbors multiple response elements to these sex hormones (*13*–*18*, *59*). After induction, GREB1 can further enhance the output of ER and androgen receptor (AR) signaling via multiple mechanisms, including direct protein interaction and enhanced ER and AR transcription activity, or enhanced ER protein stability (*13*–*14*, *18*). Accordingly, GREB1 plays a pivotal role in those hormone-stimulated cell growth and transformation, including ER*^+^* breast cancer, ovarian cancer, and prostate cancer, as well as endometrial stromal decidualization (*13*–*18*, *59*). Given this well-documented hormone-related function of GREB1, the discovery of strong GREB1 expression in *MNA^+^* NB is unexpected. Although we did not observe a high frequency of coamplification of *GREB1* and *MYCN*, we find that these two genes are strongly coexpressed in NB cell lines and patient specimens. We speculate that local chromosomal architecture changes due to the adjacent *MYCN* amplicon may also activate *GREB1* transcription, e.g., via enhancer hijacking by the *MYCN* amplicon that is known to affect adjacent genes in *MNA^+^* NB (*12*), which warrants further investigation. Moreover, the extremely high levels of transcripts encoded within the *MYCN* amplicon, including *MYCN* itself, can potentially mediate “sponge effects” of repressive gene regulators such as microRNAs (*7*). Therefore, an intriguing possibility is that competitive endogenous RNA (ceRNA) effects by transcripts encoded within the *MYCN* amplicon may also drive *GREB1* expression in *MNA^+^* NB, although our data do not suggest a direct role of *MYCN* mRNA itself in this regulation. Moreover, high *GREB1* expression may not be exclusive to *MNA^+^* NB, as occasional *MNA*^−^ NBs also express high *GREB1* levels (e.g., NB69 cells), likely through distinct mechanisms and with oncogenic functions, such as is described in ER*^+^* breast cancers and prostate adenocarcinomas (*13*–*16*).

To better understand the consequences of GREB1 OE in *MNA^+^* NB, we used a bioinformatic approach to cross-reference multiple publicly available or in-house generated RNA-seq, and proteomic datasets, including transcriptomes of two large NB tumor cohorts, RNA-seq of NB cells ± *GREB1* depletion or *MYCN* depletion, and an integrated translatomic and proteomic screen for suppressors of *anoikis* in transformed cells. This demonstrated that GREB1 transcriptionally activates *MYO1B* expression, potentially explaining why MYO1B is overexpressed in *MNA^+^* NB. Having said this, our RNA-seq performed on NB cells with or without *GREB1* depletion (Fig. 2, A and B) revealed a newly identified 388 potential “GREB1-signature genes in *MNA^+^* NB” (Fig. 2Civ), suggesting that MYO1B may be only one of a larger set of functional mediators of GREB1 in NB. It is known that MYO1B protein controls biological processes associated with actin and cell membrane dynamics, such as filopodia formation, axon formation, intracellular secretory granule formation at the trans-Golgi region, and protein transport within multi-vesicular sorting endosomes (*24*–*27*, *42*, *45*). To our knowledge, ours is the first study to systematically characterize the role of MYO1B in regulating the secretion of tumor cell contents into the extracellular space. In support of this function, we observed a marked presence of MYO1B in membrane/vesicle structures budding off the plasma membrane of tumor cells, not only in NB cells but also in murine fibroblasts transformed by *KRas^G12V^* or EN oncogenes. These findings suggest that MYO1B might be commonly exploited by diverse tumor cells in the control of secretome activity. Although we have not determined the exact mechanism mediating secretome regulation by MYO1B, we speculate that as a myosin family protein that mediates mechanobiological force generation (*60*), MYO1B may exploit this activity during budding structure formation before protein secretion to enhance secretion. In addition to *MNA^+^* NB, we also found markedly increased *MYO1B* expression in a range of other human cancers including cervical, colon, esophageal, head and neck, rectal, stomach, testicular, melanoma, thymoma, and diffuse large B cell lymphoma, as compared with their respective normal control tissues. We speculate that control of the secretome by MYO1B may have far-reaching effects beyond tumor cell–intrinsic functions, since tumor-derived secreted factors play fundamental roles in reshaping the tumor microenvironment, such as by driving immune evasion and priming the formation of premetastatic niches (*61*–*62*). Thus, MYO1B may play a crucial role in the pathobiology and malignant progression across diverse cancers, which remains unexplored.

In *MNA^+^* NB cells, this newly defined function of MYO1B in secretome regulation contributes to their high invasive and metastatic capacity. Specifically, among the large number of secreted proteins under MYO1B control, we validated that the cytokine MIF is an important functional mediator (albeit unlikely to be the only one) of MYO1B, through enhanced MIF secretion. MIF OE has been reported in diverse cancers including NB, where it has been shown to enhance tumor growth, invasiveness, and angiogenesis (*33*, *35*, *63*). Mechanistically, secreted MIF activates ERK1/2, SRC, and AKT signaling pathways via surface receptors, including CD74, CD44, and chemokine receptors CXCR2/4/7 (*33*, *35*). Here, our study uncovers that MIF secretion is controlled by MYO1B, which provides a mechanism of action as to how MIF is deployed by cancer cells for aggressive progression. Nevertheless, we suspect that, in addition to MIF, various other secreted proteins in MYO1B-controled secretome likely work in concert to promote NB tumor progression.

On the basis of the strong correlation between MYCN and MYO1B levels, we originally speculated that MYCN controls *MYO1B* expression, but instead found that GREB1 drives *MYO1B* expression. This observation does not appear to be unique to *MYO1B*. For example, despite the high correlations observed between the expression of *MYCN* and *eEF2K* and *LMO1* genes, which have shown to be important drivers of NB cell survival and metastasis, respectively, MYCN does not appear to directly regulate their expression (*64*–*65*). Therefore the present study supports the notion that, despite being a defining oncogenic driver in *MNA^+^* NB, MYCN itself does not dictate all known molecular features in this high-risk subgroup of NB. Specifically, *GREB1*, which is coexpressed with but not regulated by *MYCN*, also contributes to the pathobiology of *MNA^+^* NB, specifically through a previously unknown mechanism involving MYO1B-mediated MIF secretion to enhance the aggressiveness of *MNA^+^* NB independently of MYCN. Moreover, our evidence suggests that *MIF*, unlike *MYO1B*, is likely a direct downstream transcriptional target of MYCN (see fig. S10, A to D). This points to a collaboration between MYCN, which induces *MIF* expression, and MYO1B, which then acts in concert to promote MIF secretion. This finding suggests that *MNA^+^* NB can exploit an intricate network that involves both MYCN-dependent and MYCN-independent mechanisms during NB tumor progression.

## MATERIALS AND METHODS

### Cell lines

NIH3T3, HEK293T, BE2C, and SHEP were purchased from the American Type Culture Collection. Kelly cells were purchased from Sigma-Aldrich. NB-19, SK-N-FI, and CHLA-90 cells were a gift from Y. DeClerck (Children's Hospital Los Angeles, Los Angeles, USA). NB-SD, NMB, NB69, and SK-N-SH cells were obtained from the Children’s Hospital of Philadelphia (CHOP) cell line bank. Tet21N cells were a gift from M. Schwab (German Cancer Research Center, Heidelberg, Germany). NIH3T3 cells stably expressing the empty vector Murine Stem Cell Virus (MSCV), oncogenic EN (ETV6-NTRK3), or mutant KRas^V12^ were maintained in Dulbecco’s modified Eagle’s medium (DMEM; Sigma-Aldrich) with 10% bovine serum (calf serum, Gibco). All cell lines have been authenticated by short tandem repeat (STR) profiling using the AmpFLST Identifiler PCR Amplification Kit (Applied Biosystems) and were tested for mycoplasma on a regular basis using the LookOut Mycoplasma Detection Kit (Sigma-Aldrich). HEK293T cells were cultured in DMEM with 10% fetal bovine serum (FBS; Gibco). All other cell lines were cultured in RPMI 1640 supplemented with 10% FBS. All media were supplemented with 1% antibiotic-antimycotic (Gibco), and all cells were cultured at 37°C with 5% CO_2_.

### Plasmids and stable transfection

The LV-pEf1-tdT-luc2-WRPE lentiviral plasmid that was used to generate stable cells expressing tdTomato and firefly luciferase was a gift from J. Ronald (Western University, Canada). The pEGFPc1-*MYO1B* plasmid was a gift from E. Coudrier (Institut Curie, Centre de Recherche, Paris, France). For stable gene KD, pLKO.1 lentiviral shRNA constructs were obtained from Sigma-Aldrich MISSION shRNA consortium, including sh*MYCN* #1 (TRCN00000358381), sh*MYCN* #5 (TRCN0000020695), sh*MYCN* #6 (TRCN0000020696), sh*MYCN* #7 (TRCN0000020697), sh*Myo1b* #66 (TRCN0000100866), sh*Myo1b* #67 (TRCN0000100867), and nontargeting control shRNA (SHC002). Stable cell lines were generated by lentiviral transduction. Briefly, to generate lentiviral particles, HEK293T cells were transfected with the above plasmids together with lentiviral envelope and packaging constructs pVSVG and psPAX2 with Lipofectamine 2000 (Invitrogen) following the manufacturer’s protocol. The lentiviral particles were collected 2 to 3 days after transfection and filtered through 0.45-μl pores before cell transduction with polybrene (10 μg/ml) (Santa Cruz Biotechnology). The cells were selected with puromycin 72 hours after transduction, and nontransduced cells were used as negative controls for stable clone selection.

### siRNAs and transfection

All siRNAs were purchased from Dharmacon, including human *MIF* siRNA #11 (J-011335-11), #13 (J-011335-13), and SMARTPool *MIF* siRNA (L-011335-00), human *MYO1B* siRNA #9 (J-023110-09), #11 (J-023110-11), #12 (J-023110-12), and SMARTPool *MYO1B* siRNA (L-023110-01), human *GREB1* SMARTPool siRNA (M008187-01-0005), human *DDX1* SMARTPool siRNA (M011993-00), human *NBAS* SMARTPool siRNA (M020986-00), human *MYC* siRNA #16 (D-003282) and #35 (D-003282-35), and mouse *Myo1b* siRNA #11 (J-045103-11) and #12 (J-045103-12). Nontargeting siRNA (D-001810-10, Dharmacon) was used as a negative control. All siRNAs were transfected at a final concentration of 30 to 50 nM using Lipofectamine RNAiMAX transfection reagent (Invitrogen) and Opti-MEM I Reduced Serum Medium (Gibco) following the manufacturer’s protocol. For six-well plates, 8 μl of transfection reagent was used per well, and the same ratio was used across different formats of plates.

### RNA-seq analysis

Kelly cells transfected with 30 nM si*GREB1* or siCtrl for 48 hours (before the onset of major cell death induced by *GREB1* KD) were used for the RNA-seq analysis. Briefly, ~1 × 10^6^ cells were reconstituted in TRI Reagent (Zymo Research) and RNA was extracted using the Direct-zol RNA Microprep Kit (Zymo Research) with the optional on-column deoxyribonuclease I digest according to the manufacturer’s instructions. Extracted RNA was prepared for RNA-seq using the MGIEasy RNA Directional Library Preparation Kit with the MGI rRNA Depletion Kit according to the manufacturer’s instructions. Prepared samples were multiplexed during adapter ligation using the MGIEasy DNA Adapters provided with the RNA kit. Multiplexed samples were sequenced with 150–base pair paired-end reads on a DNBSEQ-G400 sequencer (MGI). Resulting fastq files were processed using BBDuk (ktrim = r k = 23 mink = 11 hdist = 1tpe tbo) to remove any adapter sequences and low-quality bases. For quantification, BBDuk processed files were processed using Salmon (version 1.5.2) (*66*) using selective alignment (--validateMappings) with a decoy-aware transcriptome based on the full genome (GRCh38, Gencode release 38) and GC bias correction (--gcBias). Quantification data were further parsed in R using the tximport (*67*) and DESeq2 (*68*) packages to facilitate comparisons between sample sets.

### Ex ovo chick embryo CAM model

Tumor cell extravasation assay using the chicken CAM model was performed as previously described (*50*). Briefly, Kelly or SHEP cells stably expressing tdTomato and firefly luciferase were dissociated with trypsin and counted. After washing three times with phosphate-buffered saline (PBS), the cells were resuspended to 1 × 10^6^ to 2 × 10^6^ cells/ml, and 100 μl of the cell suspension, i.e., 100,000 cells for Kelly and 200,000 cells for SHEP, was injected intravenously into each embryo. About 2 hours after injection, cells in the vasculature were counted in a marked region of the CAM. Twenty-four hours after injection, extravasated cells in the marked region were counted and extravasation rates were quantified. For the tumor metastasis assay using the chick CAM model, Kelly or SHEP cells stably expressing tdTomato and firefly luciferase were dissociated with trypsin and counted. After washing three times with PBS, the cells were resuspended to 1 × 10^6^ cells/ml, and 200 μl of the cell suspension, i.e., 200,000 cells, was injected intravenously into each embryo. Seven days after injection, embryos were sacrificed and organs were removed. Organs were injected with luciferin, incubated for 5 min, and imaged with IVIS Lumina (Caliper Life Sciences, Waltham, MA) using the bioluminescent intensity (BLI) optical imaging setting for 3 min. A region of interest (ROI) was drawn around each organ, and total flux (photons/second) was measured for each ROI. For subsequent histopathological analyses, the tissues were formalin-fixed overnight and stored in 70% ethanol before being embedded in paraffin.

### Immunoblotting analysis

Immunoblotting analysis was performed following standard protocols using 10% or 12% SDS–polyacrylamide gel electrophoresis (PAGE) gels, as described (*40*). Briefly, 10 to 30 μg of protein lysates were loaded per well, and nitrocellulose membranes (Bio-Rad) were used for protein transfer. The primary antibodies used were MYO1B (Sigma-Aldrich, HPA013607), MYO1B (Abcam, ab194356), GREB1 (Millipore, MABS62), MYCN (Abcam, ab119701), c-MYC (Cell Signaling Technology, #9402), glyceraldehyde-3-phosphate dehydrogenase (GAPDH) (Cell Signaling Technology, #5174), and β-actin (Cell Signaling Technology, #8457). Horseradish peroxidase (HRP)–conjugated secondary antibody goat anti-rabbit immunoglobulin G (IgG) (Cell Signaling Technology) was used. Membranes were developed using Pierce ECL Western Blotting Substrate (Thermo Fisher Scientific). Images were acquired with ImageQuant LAS4000 Luminescent Image Analyzer (GE).

### Immunohistochemistry

An NB FFPE (formalin-fixed paraffin-embedded) tissue microarray we recently described (*64*) was used in this study. MYO1B and NCAM expression in the FFPE tissues was assessed using a Ventana DISCOVERY Ultra autostainer (Ventana Medical Systems, Tucson, AZ). For MYO1B IHC, baked and deparaffinized FFPE tissue sections were first incubated in tris-based buffer (CC1, Ventana) at 95°C for 1 hour for antigen retrieval, followed by incubation at room temperature for 1 hour with MYO1B antibody (1:500, Abcam, ab194356) or NCAM1 antibody (1:200, Abcam, ab133345) prepared in DISCOVERY Ab diluent (Ventana). Bound primary antibodies were amplified with AffiniPure Goat Anti-Rabbit IgG (H+L) (1:500, Jackson ImmunoResearch, rabbit polyclonal, catalog no. 111-005-003) and visualized with the UltraMap DAB anti-Rb Detection Kit (Ventana). For IHC scoring, staining intensity was assigned via a four-point scale system (0 = no staining, 1 = low, but detectable degree of staining, 2 = clearly positive cases, and 3 = strong expression) and percentage of positive cells (0 to 100%) was also determined. IHC H-score was then calculated per sample as staining intensity multiplied by percentage of positive cells. For analysis of tissue microarrays (TMAs), the average IHC score was calculated among duplicate tissue cores from the same patient.

### IF analysis

Cells grown on chamber slides (Millipore) were fixed in 4% paraformaldehyde for 10 min at room temperature and rinsed three times in PBS between each of the following steps. Cells were permeabilized and blocked in PBS containing 0.2% Triton X-100 and 2% bovine serum albumin (BSA) for 30 min at room temperature. Then, cells were incubated at room temperature for 1 hour with primary antibodies against MYO1B (1:500, ab194356, Abcam or 1:200, HPA013607, Sigma-Aldrich), MIF (0.35 μg/ml, AF-289, R&D Systems), and GREB1 (1:200, #65171, Cell Signaling Technology) diluted in PBS containing 2% BSA. Cells were subsequently incubated at room temperature for 1 hour with respective IgG (H+L) secondary antibodies conjugated with Alexa Fluor 488 and/or Alexa Fluor 568, and/or Alexa Fluor 594, and/or Alexa Fluor 647 (Molecular Probes, all 1:200), all diluted in PBS containing 2% BSA. For actin cytoskeleton staining, tetramethyl rhodamine isothiocyanate (TRITC)–phalloidin (1 to 2 μg/ml final concentration, Millipore) was coincubated with the above secondary antibodies. Then, cells were rinsed with PBS three times, and nuclei were counterstained with DAPI (4′,6-diamidino-2-phenylindole) within the VECTASHIELD Hardset Antifade Mounting Medium (Vector Laboratories). Images were taken on an LSM Airyscan 800 confocal microscope using a 63× oil immersion objective and the Zen Blue software (Zeiss). To compare protein expression levels in a specific panel of cell lines, the same parameters were used across the samples being compared for both image capturing and processing.

### ELISA analysis

MIF concentrations in CM were measured by ELISA assays using a human MIF ELISA kit (RAB0360, Sigma-Aldrich), according to the manufacturer’s instructions. To collect CM for ELISA analysis, equal amounts of cells, i.e., 5 × 10^5^ cells per well in six-well plates, were cultured in 2 ml of RPMI growth medium containing 10% FBS for 24 hours before CM collection. Then, cell debris in the CM samples were cleaned up via two sequential centrifugations at 300*g* for 10 min and 3000*g* for 10 min. The CM samples were either used directly in ELISA analysis or processed as follows: (i) lysed with equal amount of RIPA buffer for 30 min on ice with sonication (60% power, three cycles, 10 s per cycle), (ii) filter the CM through filters with 20-nm pores (Sigma-Aldrich) and collect the filtrate for ELISA analysis, and (iii) filter the CM through columns with 100 kDa cutoff (Millipore) and collect the filtrate for ELISA analysis. To generate negative controls, fresh RPMI growth medium was processed with the above procedures. All the CM samples were diluted 10× with double-distilled water for ELISA measurements.

### Cell migration and invasion assays

Cells transfected with siRNAs (for 48 hours) were plated in 12-well plates containing ibidi four-well culture inserts (ibidi) that create wounds, which were mounted with 25% Matrigel (Corning) diluted in growth medium. Cell invasion through the Matrigel was evaluated by measuring wound closure distance at different time points by ImageJ software. Alternatively, cells were seeded into a 96-well plate, 8 × 10^4^ cells per well. Scratches were created using a wound maker (ESSEN), and the wells were rinsed with culture medium once to remove cell debris. After removing the medium, the wounds were mounted either with fresh growth medium (for migration assay) or with 25% Matrigel (60 μl per well) (Corning) diluted in growth medium. For rMIF treatment (catalog no. 289-MF, R&D Systems), rMIF was added to the Matrigel at 0, 20, or 100 ng/ml. Then, 150 μl of RPMI medium containing 0, 20, or 100 ng/ml was added to each well when the Matrigel gets solidified after about 3 hours. Incucyte was used to monitor and quantify cell invasion, as measured by the distance of cells invading through Matrigel mounted on top of the wound.

### Cell growth, cell death, and anoikis analysis

Cell growth was analyzed by the Incucyte system with 2- to 4-hour scanning intervals, two fields per well were imaged, and three to eight wells were imaged per condition. Briefly, cells were plated at 2000 to 5000 cells per well in 96-well plates, and growth curves were generated based on the percentage of cell confluence automatically detected and quantified by the Incucyte software. Cell death was determined by SYTOX Orange nucleic acid stain (Invitrogen). Briefly, the reagent was added to the culture medium at a concentration of 0.5 to 1 μM and the fluorescent signals were monitored and analyzed by Incucyte analysis. Anoikis assay was performed by plating cells in round-bottom ultralow attachment 96-well plates (Corning), 2000 to 10,000 cells per well, and the cells were cultured in normal cell growth medium. To determine anoikis, 0.5 μM SYTOX Orange nucleic acid stain (Invitrogen) was added to the culture medium and the fluorescent signals were monitored and analyzed by Incucyte with 4- to 8-hour scanning intervals, and three to eight wells were imaged per condition. To calculate cell death or *anoikis* index, cell confluence (2D cultures) and spheroid area (3D cultures) determined by the Incucyte were used to normalize the SYTOX Orange intensity. To directly compare absolute cell death rates in 2D and anoikis in 3D conditions, flow cytometry was performed after staining the cells with propidium iodide (PI). Briefly, siRNA-transfected cells (at 24 hours) were plated into regular six-well plates or flat ultralow attachment six-well plates (Corning), 100,000 cells per well. After 4 days of culture in normal cell growth medium, cells in 2D plates and spheroids were gently dissociated using Cellstripper (Corning), a mild nonenzymatic cell dissociation solution, and all cells were collected for cell death analysis. For the PI staining, the dissociated cells were first fixed for 2 hours on ice in 70% cold ethanol prepared with water. The cell pellets were collected by centrifugation at 850*g* for 5 min and washed with cold PBS twice. Then, the cells were stained with PI (50 μg/ml) in PBS containing ribonuclease A (200 μg/ml) (Thermo Fisher Scientific) and 0.1% Triton X-100 for 15 min in a 37°C water bath. Finally, the cells were resuspended in cold PBS and analyzed by flow cytometry, and the percentage of cells with hypodiploid DNA contents were analyzed by FlowJo software.

### Bioinformatic analysis

GREB1 ChIP-seq data in breast cancer MCF7 cells were extracted from Gene Expression Omnibus GSE41561 (*13*). Gene expression changes of *GREB1* and *MYO1B* upon stable *GREB1* KD in prostate adenocarcinoma LNCaP cells were derived from RNA-seq analysis deposited in Gene Expression Omnibus GSE120720 (*18*). Transcriptome data of Tet21N NB cells with *MYCN* gene depletion by doxycycline treatment were extracted from Gene Expression Omnibus GSE80153 (*41*). Genomics data on gene copy numbers, including *MYCN*, *DDX1*, *NBAS*, *GREB1*, *FAM49A*, and *FAM84A*, in 554 NB tumors (*69*) and 31 NB cell lines were extracted from R2: Genomics Analysis and Visualization Platform (http://r2.amc.nl) and DepMap project (https://depmap.org/portal/), respectively. *MYO1B* expression data in pediatric cancers from the TARGET project cohort were extracted from cBioPortal (https://www.cbioportal.org/). Transcriptome data, including genes that are positively correlated with MYCN and GREB1 expression in NB patient samples, mRNA expression levels of *MYO1B*, *MYCN*, *MIF*, and *GREB1* in multiple NB, MB, and prostate cancer cohorts were extracted from R2: Genomics Analysis and Visualization Platform. Kaplan-Meier survival curves for NB patients were generated using R2: Genomics Analysis and Visualization Platform, and the optimal cutoff was automatically determined by the platform. The expression profiles of *GREB1* and *MYO1B* mRNA in human cancer cell lines were acquired from the DepMap project. To compare *MYO1B* expression in various tumor versus normal tissues, the plots were generated using data from GEPIA: Gene Expression Profiling Interactive Analysis database (http://gepia.cancer-pku.cn).

### Quantification and statistical analysis

Statistical details of MS data analysis are described separately in the section devoted to this type of data acquisition. GraphPad Prism software (version 8) was used for all other statistical analyses. Student’s unpaired *t* tests (two-tailed) were used to compare differences between two groups. Pearson correlation coefficients were calculated to determine the correlation between two groups. Fisher’s exact test was used to determine the associations between two categorical variables in two groups. Log-rank test was used in Kaplan-Meier survival analysis. For all statistical analysis, n.s., nonsignificant; *P < 0.05; **P < 0.01; ***P < 0.001. Otherwise indicated in the figure legends, all data presented are means ± SD.

## Supplementary Materials

### This PDF file includes:

Supplementary Materials and Methods Figs. S1 to S12 Legends for tables S1 to S14 References
