# Supplementary material for: A MYCN-independent mechanism mediating secretome reprogramming and metastasis in MYCN-amplified neuroblastoma
Source: Sci Adv. 2023 Aug 23;9(34):eadg6693. doi: 10.1126/sciadv.adg6693 (PMC10446492; doi:10.1126/sciadv.adg6693)
Supplement: Supplementary file 1 — Supplementary Materials and Methods Figs. S1 to S12 Legends for tables S1 to S14 References [file sciadv.adg6693_sm.pdf]

Science Advances
AAAS

Supplementary Materials for

A MYCN-independent mechanism mediating secretome reprogramming and metastasis in *MYCN*-amplified neuroblastoma

Hai-Feng Zhang *et al.*

Corresponding author: Poul H Sorensen, psor@mail.ubc.ca

Sci. Adv. **9**, eadg6693 (2023)
DOI: 10.1126/sciadv.adg6693

The PDF file includes:

Supplementary Materials and Methods
Figs. S1 to S12
Legends for tables S1 to S14
References

Other Supplementary Material for this manuscript includes the following:

Tables S1 to S14

SUPPLEMENTAL MATERIALS AND METHODS

mRNA isolation, cDNA synthesis and qPCR analysis

Total RNA was extracted from cells using the RNeasy RNA extraction kit (Qiagen) following the manufacturer's instructions. Briefly, cells were harvested in RLT buffer directly from cell culture plates. Genomic DNA was removed from the lysates using genomic DNA removal columns (supplied by the kit) before isolating total RNA. cDNA was generated from 500-1000ng of total RNA using the High-Capacity cDNA Reverse Transcription kit (Applied Biosystems). qPCR analysis was performed using Fast SYBR Green Master Mix (Applied Biosystems) in a QuantStudio 6 Real-Time PCR Systems instrument (Thermo Fisher). The primers used are listed in the qPCR primers table below. The reactions were performed in 384-well plates with 40 thermal cycles. For qPCR data analysis, ΔΔCt method (Applied Biosystems) was used for normalization to an endogenous control (*ACTB* or *GAPDH*) and calculation of gene expression changes.

qPCR primers

**qPCR primers**	**Forward (5′-3')**	**Reverse (5′-3')**
MYCN (human)	AGCGATTCAGATGATGAAGA	GTGATGGTGAATGTGGTGACA
MYO1B (human)	GGGCTTACTGGCTTGGATCT	ACAGCAACTGCATGCTTACG
GREB1 (human)	GGACAGAAGGCTCTACCTC	AGCGCTGAACCGGAAGCCTT
NBAS (human)	GCAGACAGAAATTCTTTATCA	CTACTTCATCCTCTTGTACT
DDX1 (human)	CCACATTAGAACTGATGATG	GACCACATCTCTGGACTATT
MIF (human)	GGTTCCTCTCCGAGCTCA	ACCACGTGCACCGCGATGTA
MYC (human)	TCGGATTCTCTGCTCTCCTC	TCGGTTGTTGCTGATCTGTC
ACTB (human)	AGAGCTACGAGCTGCCTGAC	AGCACTGTGTTGGCGTACAG
GAPDH (human)	GAAGGTGAAGGTCGGAGTCA	TTGAGGTCAATGAAGGGGTC

Cleavage Under Targets and Release Using Nuclease (CUT&RUN) analysis

The CUT&RUN analysis[44] was performed using the CUTANA™ ChIC/CUT&RUN kit from EpiCypher (#14-1048) according to the manufacturer's instructions with the following modifications. Briefly, Kelly cells were plated into a 6-well plate, 200,000 cells/well. The next day, the cells were moderately cross-linked for 1min directly in the culture media with 1% formaldehyde (Electron Microscopy Sciences, #15714-S). Then, the formaldehyde was quenched by adding 125mM glycine. The cells were then washed in plate with cold PBS twice, and the nuclei were isolated using a nuclei isolation kit from Sigma (#NUC-101). The nuclei were resuspended in wash buffer provided by the kit and were counted. Then, for each CUT&RUN reaction, 500,000 nuclei and 2μg antibodies were used, including GREB1 antibody

(Millipore, MABS62) or the isotype control normal mouse IgG (Santa Cruz, sc-2025). The rest of the steps were conducted strictly according to the manufacturer's instructions, and 12µl elusion buffer was used to elute the final DNA product from the CUT&RUN reactions. To validate GREB1 binding to the *MYO1B* locus, qPCR was performed using the CUT&RUN DNA (with 7x dilution) and primers spanning a predominant peak identified in a previous GREB1 ChIP-seq analysis in MCF7 cells[13] [**Fig. S4B**]. The primer sequences are Forward 5'-GGAGCTTGTAGTTCCATCTGA-3' and Reverse 5'-CTTCTGCAGAGTTGCTTGGTA-3', and the predicted PCR product size is 203bp. The qPCR analysis was performed using Fast SYBR Green Master Mix (Applied Biosystems) according to the manufacturer's instructions in a QuantStudio 6 Real-Time PCR Systems instrument (Thermo Fisher). The qPCR reactions were performed in 384-well plates, 12µl/reaction, with 40 thermal cycles. The qPCR results were further validated by agarose gel electrophoresis (2% gel) using PCR products from 60 thermal cycles, and 0.1% input DNA was used as a positive control.

Total proteome analysis by mass spectrometry

To determine the impact of MYO1B depletion on the total proteome of *MYCN*-amp neuroblastoma cells, Kelly and NB19 cells were transfected for 72-hours with non-targeting control (siCtrl) or SMARTPool siRNAs (Dharmacon) targeting *MYO1B*. Three biological replicates (4 x 10e6 cells/condition/replicate) of the cells were harvested by trypsinization, and rinsed with cold PBS prior to freezing. Efficient reduction of MYO1B protein was validated by immunoblotting prior to mass spectrometry (MS) sample preparation. Samples were prepared for MS analysis as described previously[40]. Briefly, cell lysis was performed using a solution of 100mM HEPES pH 8.5, 2% SDS, 50mM NaCl, 1X cOmplete protease inhibitor cocktail, and 10mM dithiothreitol (DTT). Cell lysates were disrupted using Lysing Matrix Y tubes in a FastPrep instrument (6M/s, 2 cycles, 45s per cycle). Lysates were incubated for 30-minutes at 60°C with mixing at 1,000rpm in a Thermomixer. To the incubated lysates, 20mM iodoacetamide (IAA, final concentration) was added, and the samples incubated at 24°C for 30-minutes in the dark. Reactions were then quenched with the addition of 10mM of DTT (final concentration). Proteins were purified from the prepared lysates prior to proteolysis using SP3, as described previously[70-71]. A 10µL volume of the prepared SP3 carboxylate bead stock was added to each lysate and absolute ethanol added to a final concentration of 50% by volume. Reactions were incubated for 10-minutes at room temperature with periodic mixing prior to centrifugation for 5-minutes at 5,000g. On a magnetic rack, the unbound supernatant was removed to waste and the beads rinsed 3x off-rack with 800µL of 80% ethanol. For typtic

digestion, rinsed beads were reconstituted in 100mM HEPES pH 8 containing a mixture of trypsin and rLysC (Promega) at an approximate concentration of 1:50 (µg:µg, trypsin to protein) in a volume of 100µL per sample. Digestion was carried out at 37°C with mixing at 1,000rpm for 18-hours in a Thermomixer. Digested samples were centrifuged at 12,000g for 2-minutes and placed on a magnetic rack to recover the peptide-containing supernatant.

After digestion, tryptic peptides were labeled with 10-plex tandem mass tags (TMT, Thermo Scientific). Specifically, TMT labels from the vendor were reconstituted at a concentration of 10mg/mL in acetonitrile (HPLC grade, Thermo Scientific). Peptides were labeled with two separate additions of TMT label (each addition at a volume equivalent to 2:1, µg:µg, TMT label to peptide), 20-minutes apart with incubation at 24°C. TMT reactions were quenched using a volume equivalent to the amount of added TMT label from a 1M solution of glycine. TMT labeled samples were evaporated to dryness using a SpeedVac centrifuge and subsequently reconstituted in 0.1% trifluoroacetic acid (TFA) into a single multiplexed sample. The combined sample was desalted using a Sep-Pak cartridge (100mg tC18, Waters) using a loading series of acetonitrile + 0.1% TFA, water + 0.1% TFA, combined sample, water + 0.1% formic acid (FA), and 60% acetonitrile + 0.1% FA. Eluted peptides were evaporated to dryness using a SpeedVac centrifuge and subsequently reconstituted in 20mM ammonium bicarbonate (pH 8) prior to fractionation. High-pH C18 reversed phase fractionation was performed on an Agilent 1100 HPLC system equipped with a diode array detector (254, 260, and 280nm) and a Kinetix EVO C18 column (2.1 x 150mm, 1.7 µm core shell, 100Å, Phenomenex). Elution was performed at a flow rate of 0.2mL per minute using a gradient of mobile phase A (10mM ammonium bicarbonate, pH 8) and B (acetonitrile), from 3% to 45% over 60-minutes. Fractions were collected every minute across the elution window for a total of 48 fractions, which were concatenated to a final set of 12 (e.g. 1 + 13 + 25 + 37 = fraction 1). Fractions were dried in a SpeedVac centrifuge and reconstituted in 1% FA prior to MS analysis.

Analysis of TMT labeled peptide fractions was carried out on an Orbitrap Fusion Tribrid MS platform (Thermo Scientific). Samples were introduced using an Easy nLC 1000 system with a trapping-analytical column setup (Thermo Scientific). Trapping columns were packed in-house in 100µm internal diameter capillaries to a length of 25mm with C18 beads (Reprosil-Pur, Dr. Maisch, 3µm particle size). Trapping was carried out for a total volume of 10µL at a pressure of 400bar. After trapping, gradient elution of peptides was performed on a C18 (Reprosil-Pur, Dr. Maisch, 1.9µm particle size) column packed in-house

to a length of 25cm in 100μm internal diameter capillaries with a laser-pulled electrospray tip and heated to 45°C using AgileSLEEVE column ovens (Analytical Sales & Service). Elution was performed with a gradient of mobile phase A (water and 0.1% formic acid) and B (acetonitrile and 0.1% formic acid) over 60-minutes at a flow rate of 400nL/min.

Data acquisition on the Orbitrap Fusion was carried out using a data-dependent method with multi-notch synchronous precursor selection MS3 scanning for TMT tags. Survey scans covering the mass range of 380 – 1500 m/z were acquired at a resolution of 120,000 (at m/z 200), with quadrupole isolation enabled, an S-Lens RF Level of 60%, a maximum fill time of 30 milliseconds, and an automatic gain control (AGC) target value of 4e5. For MS2 scan triggering, monoisotopic precursor selection was enabled (Peptide mode), charge state filtering was limited to 2 – 4, an intensity threshold of 5e3 was employed, and dynamic exclusion of previously selected masses was enabled for 45-seconds with a tolerance of 20 ppm. MS2 scans were acquired in the ion trap in Rapid mode after CID fragmentation with a maximum fill time of 30 milliseconds, quadrupole isolation, an isolation window of 1 m/z, collision energy of 30%, activation Q of 0.25, injection for all available parallelizable time turned OFF, and an AGC target value of 1e4. Fragment ions were selected for MS3 scans based on a precursor selection range of 400-1600 m/z, ion exclusion of 20 m/z low and 5 m/z high, a relative intensity threshold of 10, and isobaric tag loss exclusion for TMT. The top 10 precursors were selected for MS3 scans that were acquired in the Orbitrap after HCD fragmentation (NCE 60%) with a maximum fill time of 120 milliseconds, 50,000 resolution, 120-750 m/z scan range, ion injection for all parallelizable time turned OFF, and an AGC target value of 4e5. The total allowable cycle time was set to 4 seconds. MS1 and MS3 scans were acquired in profile mode and MS2 in centroid format.

Secretome analysis by mass spectrometry

To purify proteins secreted by cells into conditioned medium (CM), a combination of pulsed azidohomoalanine (AHA) and SILAC (stable isotope labeling with amino acids in cell culture) was used as described previously[40,53-54]. As in the total proteome analysis, Kelly and NB19 cells transfected with control or *MYO1B* siRNAs in biological triplicate. Forty-eight hours after transfection, the cells were incubated for on hour in 10% FBS containing-medium lacking methionine, arginine, and lysine (Caisson Labs) prior to pulsed-AHA and -SILAC treatment. Then, Kelly and NB19 siCtrl cells were labeled with medium ($^{13}C_6$ arginine, $^{2}H_4$ lysine) versions of arginine and lysine, and AHA (100μM final, AnaSpec)

while si*MYO1B* cells were incubated with heavy ($^{13}C_6$ $^{15}N_4$ arginine, $^{13}C_6$ $^{15}N_2$ lysine) versions of arginine and lysine and AHA. The cells were pulse-labeled for an additional 24-hours, and CM samples were harvested and centrifuged for 5-minutes at 1,000g to precipitate cells and other debris. CM from siCtrl and si*MYO1B* cells were equivalently mixed and 11mL of the mixture was concentrated by filtration using an Amicon Ultra-15 centrifugal filter device (3KDa cutoff, 3,250g for 90-minutes). To isolate secreted proteins (newly synthesized proteins labeled with AHA and medium or heavy arginine and lysine), Click enrichment was performed as previously described[40] using the Click-iT Protein Enrichment Kit (Thermo Scientific). Briefly, 0.4mL of the concentrated CM was mixed with 0.45mL Urea Lysis Buffer and 800ul of this mixture was used in the Click labeling reaction according to the manufacturer's instructions. Click tagged newly synthesized and AHA labeled proteins captured using the provided resin were digested with 1μg trypsin (Promega) overnight at 37°C. After digestion, peptides were recovered and TMT labeled as described in the total proteome analysis section. TMT labeled peptides were evaporated to dryness using a SpeedVac centrifuge and subsequently reconstituted in 0.1% TFA. Samples were desalted prior to MS analysis using C18 TopTips with the same conditioning, rinsing, and elution routine as with the Sep-Pak's for total proteome analysis. MS analysis was carried out using an Orbitrap Fusion coupled to an Easy nLC 1000 as described for the total proteome analysis. For MS data acquisition, survey scans covering the mass range of 380 – 1500 m/z were acquired at a resolution of 120,000 (at m/z 200), with quadrupole isolation enabled, an S-Lens RF Level of 60%, a maximum fill time of 30 milliseconds, and an automatic gain control (AGC) target value of 4e5. For MS2 scan triggering, monoisotopic precursor selection was enabled (Peptide mode), charge state filtering was limited to 2 – 4, an intensity threshold of 5e3 was employed, and dynamic exclusion of previously selected masses was enabled for 45-seconds with a tolerance of 20 ppm. MS2 scans were acquired in the ion trap in Rapid mode after HCD fragmentation with a maximum fill time of 30 milliseconds, quadrupole isolation, an isolation window of 1 m/z, collision energy of 32%, injection for all available parallelizable time turned OFF, and an AGC target value of 1e4. The total allowable cycle time was set to 4 seconds. MS1 scans were acquired in profile mode and MS2 in centroid format.

MS data analysis

MS data were analyzed using Proteome Discoverer Software (ver. 2.1). Spectra were searched using SequestHT against Swissport human proteome database including a list of common contaminants (04/2017, 20799 sequences). Identification parameters in the SequestHT search were specified as trypsin

enzyme specificity, two missed cleavages allowed, minimum peptide length of 6, precursor mass tolerance of 20 ppm, and a fragment mass tolerance of 0.8 Daltons. Oxidation of methionine (15.995 Da), N-terminal acetylation (42.011 Da), medium (Lys4/Arg6) and heavy (Lys8/Arg10) SILAC were set as variable modifications. Carbamidomethylation of cysteine (57.021 Da) was set as a static modification. Peptide spectrum match (PSM) identification FDR was calculated using Percolator by searching the results against decoy sequences and only PSMs with FDR < 1% were retained in the analysis. Differential protein expression was calculated with PECA[72] with quantile normalization and modified t statistic as parameters, only peptides mapping to a single protein were included.

Figure S1

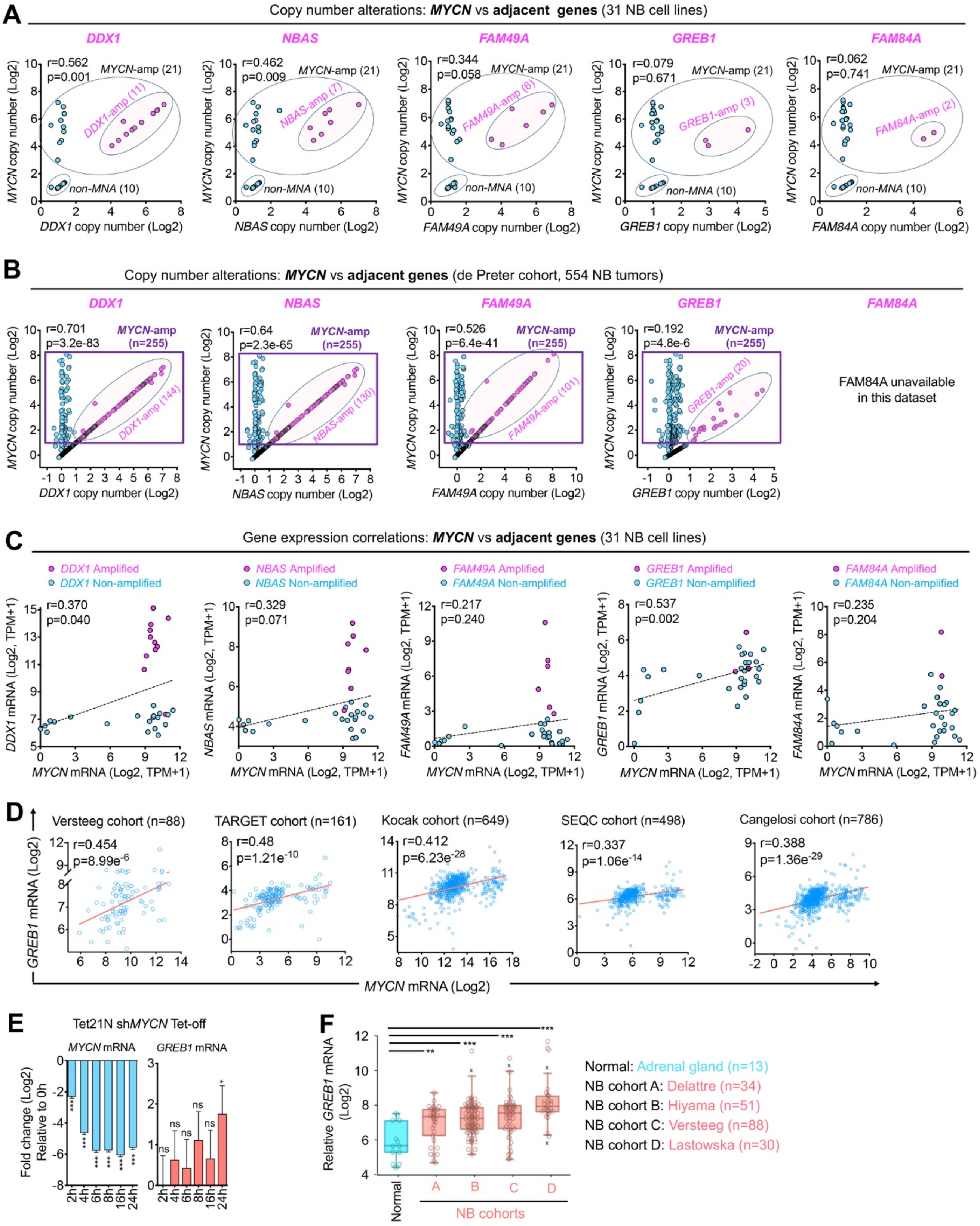
A
Copy number alterations: MYCN vs adjacent genes (31 NB cell lines)
DDX1
r=0.562
p=0.001
MYCN-amp (21)
DDX1-amp (11)
non-MNA (10)
MYCN copy number (Log2)
DDX1 copy number (Log2)
NBAS
r=0.462
p=0.009
MYCN-amp (21)
NBAS-amp (7)
non-MNA (10)
NBAS copy number (Log2)
FAM49A
r=0.344
p=0.058
MYCN-amp (21)
FAM49A-amp (6)
non-MNA (10)
FAM49A copy number (Log2)
GREB1
r=0.079
p=0.671
MYCN-amp (21)
GREB1-amp (3)
non-MNA (10)
GREB1 copy number (Log2)
FAM84A
r=0.062
p=0.741
MYCN-amp (21)
FAM84A-amp (2)
non-MNA (10)
FAM84A copy number (Log2)
B
Copy number alterations: MYCN vs adjacent genes (de Preter cohort, 554 NB tumors)
DDX1
r=0.701
p=3.2e-83
MYCN-amp (n=255)
DDX1-amp (144)
NBAS
r=0.64
p=2.3e-65
MYCN-amp (n=255)
NBAS-amp (130)
FAM49A
r=0.526
p=6.4e-41
MYCN-amp (n=255)
FAM49A-amp (101)
GREB1
r=0.192
p=4.8e-6
MYCN-amp (n=255)
GREB1-amp (20)
FAM84A
FAM84A unavailable in this dataset
C
Gene expression correlations: MYCN vs adjacent genes (31 NB cell lines)
DDX1 Amplified
DDX1 Non-amplified
r=0.370
p=0.040
DDX1 mRNA (Log2, TPM+1)
NBAS Amplified
NBAS Non-amplified
r=0.329
p=0.071
NBAS mRNA (Log2, TPM+1)
FAM49A Amplified
FAM49A Non-amplified
r=0.217
p=0.240
FAM49A mRNA (Log2, TPM+1)
GREB1 Amplified
GREB1 Non-amplified
r=0.537
p=0.002
GREB1 mRNA (Log2, TPM+1)
FAM84A Amplified
FAM84A Non-amplified
r=0.235
p=0.204
FAM84A mRNA (Log2, TPM+1)
MYCN mRNA (Log2, TPM+1)
D
Versteeg cohort (n=88)
r=0.454
p=8.99e-6
TARGET cohort (n=161)
r=0.48
p=1.21e-10
Kocak cohort (n=649)
r=0.412
p=6.23e-28
SEQC cohort (n=498)
r=0.337
p=1.06e-14
Cangelosi cohort (n=786)
r=0.388
p=1.36e-29
GREB1 mRNA (Log2)
MYCN mRNA (Log2)
E
Tet21N shMYCN Tet-off
MYCN mRNA
GREB1 mRNA
Fold change (Log2) Relative to 0h
2h
4h
6h
8h
16h
24h
ns
F
Relative GREB1 mRNA (Log2)
Normal
A
B
C
D
NB cohorts
Normal: Adrenal gland (n=13)
NB cohort A: Delattre (n=34)
NB cohort B: Hiyama (n=51)
NB cohort C: Versteeg (n=88)
NB cohort D: Lastowska (n=30)

Figure S1. Genomic alterations of genes adjacent to the MYCN locus in neuroblastoma. (A) Gene copy number correlations between MYCN and genes adjacent to the MYCN locus in 31 human NB cell lines. Data were derived from the DepMap database_Copy Number Public 22Q4. **(B)** Gene copy number correlations between MYCN and genes adjacent to the MYCN locus in a cohort of 554 NB tumors. Data were derived from the de Preter NB cohort deposited in the R2 database. **(C)** mRNA expression correlations between MYCN and various genes flanking the MYCN locus in 31 human NB cell lines. Data were derived from the DepMap database_Expression Public 22Q4. **(D)** Correlation between *MYCN* and *GREB1* mRNA expression in five cohorts of NB patient samples based on data extracted from the R2 database. **(E)** The impact of *MYCN* depletion by doxycycline on *GREB1* mRNA expression in Tet21N cells that express *MYCN* cDNA under the control of a Tet-off system. Plots were generated based on data extracted from Gene Expression Omnibus GSE80153. **(F)** Comparison of *GREB1* mRNA levels in four cohorts of NB patient samples and non-tumor adrenal gland samples, plots were derived from the R2 database. For all panels, data presented are means ± SD; p values were determined by two-tailed unpaired Student's t-test. Pearson coefficient analysis was performed to determine correlations between two variables. ns. no significance, $*p < 0.05$, $**p < 0.01$, $***p < 0.001$.

Figure S2

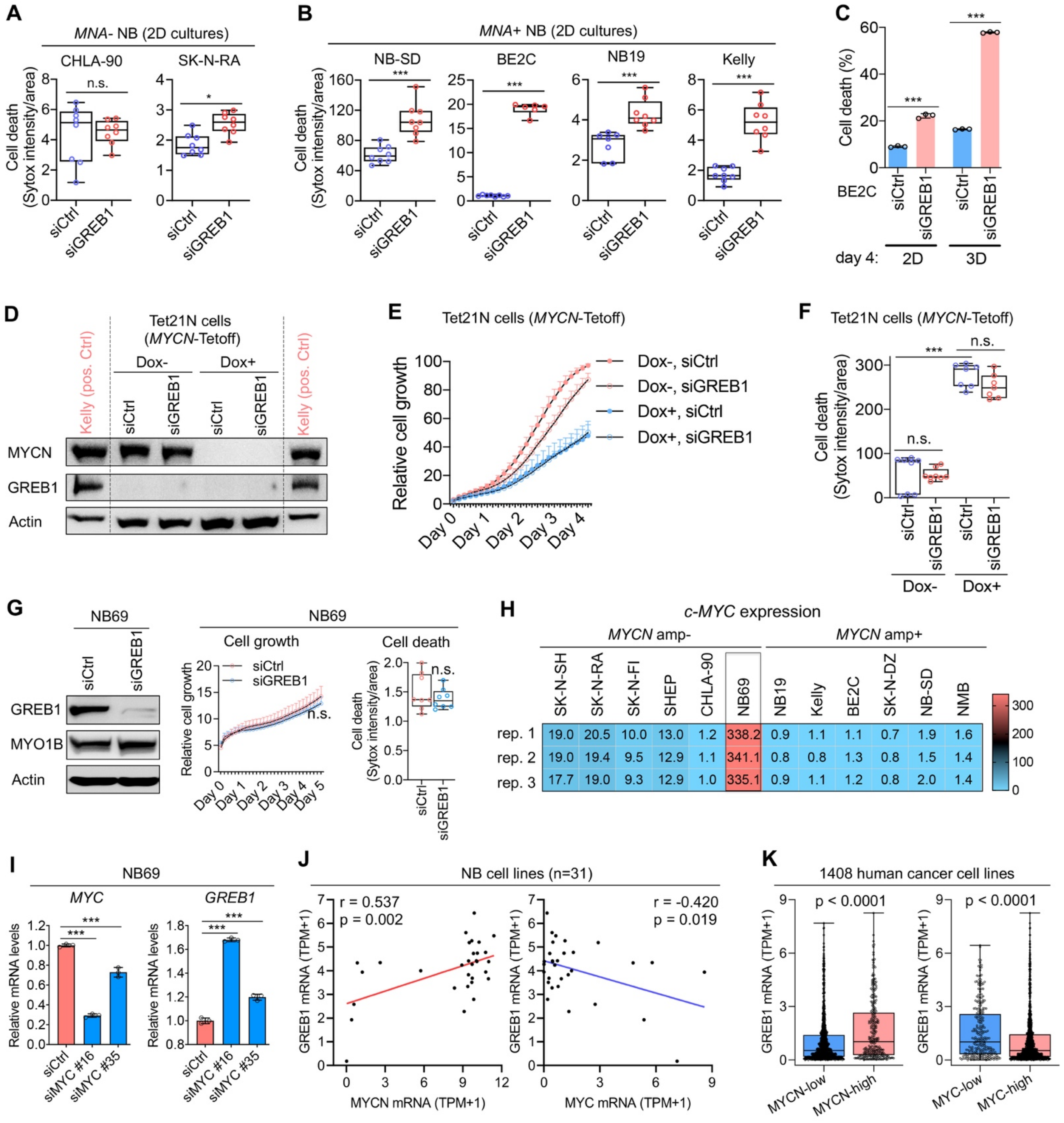

A
MNA- NB (2D cultures)
CHLA-90
SK-N-RA
n.s.
*
Cell death (Sytox intensity/area)
siCtrl
siGREB1
B
MNA+ NB (2D cultures)
NB-SD
BE2C
NB19
Kelly
***
C
Cell death (%)
BE2C
day 4:
2D
3D
D
Tet21N cells (MYCN-Tetoff)
Dox-
Dox+
Kelly (pos. Ctrl)
MYCN
GREB1
Actin
E
Tet21N cells (MYCN-Tetoff)
Relative cell growth
Dox-, siCtrl
Dox-, siGREB1
Dox+, siCtrl
Dox+, siGREB1
Day 0
Day 1
Day 2
Day 3
Day 4
F
Tet21N cells (MYCN-Tetoff)
G
NB69
Cell growth
Cell death
GREB1
MYO1B
Actin
Day 5
H
c-MYC expression
MYCN amp-
MYCN amp+
SK-N-SH SK-N-RA SK-N-FI SHEP CHLA-90 NB69 NB19 Kelly BE2C SK-N-DZ NB-SD NMB
rep. 1 19.0 20.5 10.0 13.0 1.2 338.2 0.9 1.1 1.1 0.7 1.9 1.6
rep. 2 19.0 19.4 9.5 12.9 1.1 341.1 0.8 0.8 1.3 0.8 1.5 1.4
rep. 3 17.7 19.0 9.3 12.9 1.0 335.1 0.9 1.1 1.2 0.8 2.0 1.4
I
NB69
MYC
GREB1
Relative mRNA levels
siMYC #16
siMYC #35
J
NB cell lines (n=31)
r = 0.537
p = 0.002
r = -0.420
p = 0.019
GREB1 mRNA (TPM+1)
MYCN mRNA (TPM+1)
MYC mRNA (TPM+1)
K
1408 human cancer cell lines
p < 0.0001
MYCN-low
MYCN-high
MYC-low
MYC-high

Figure S2. The role of GREB1 and MYCN in NB. **(A-B)** The impact of *GREB1* gene depletion on cell growth was evaluated by Incucyte in *MNA*- versus *MNA*+ NB cells (n=6-8). **(C)** Comparison of cell death by propidium iodide staining and flow cytometry in the indicated cells grown in 2D or 3D cultures for 4 days. **(D)** Western blotting analysis of the indicated samples. Tet21N cells were treated with 1μg/ml Dox +/- siGREB1 for 72h before the analysis. **(E)** The impact of *MYCN* and/or *GREB1* gene depletion on Tet21N cell growth was evaluated by Incucyte (n=6). **(F)** The impact of *MYCN* and/or *GREB1* gene depletion on anoikis was evaluated by Incucyte with Sytox Orange labeling in 3D spheroid cultures at day 5 (n=7-8). **(G)** Left panel, Western blotting analysis of the indicated samples. Right panel, the impact of *GREB1* gene depletion on NB69 cell growth was evaluated by Incucyte (n=8), and the impact on anoikis was evaluated by Incucyte with Sytox Orange labeling in 3D spheroid cultures at day 4 (n=8). **(H)** The expression of *c-MYC* mRNA was determined in the indicated cell lines by real-time PCR and normalized to *ACTB* house keeping gene. **(I)** The impact of *c-MYC* depletion on *GREB1* expression was determined by real-time PCR. **(J-K)** mRNA correlations between the indicated genes in 31 human NB cell lines (J) or 1408 human cancer cell lines (K). Data were derived from the DepMap database_Expression Public 22Q4. For all panels, data presented are means ± SD, p values were determined by two-tailed unpaired Student's t-test; ns. no significance, ***p < 0.001.

Figure S3

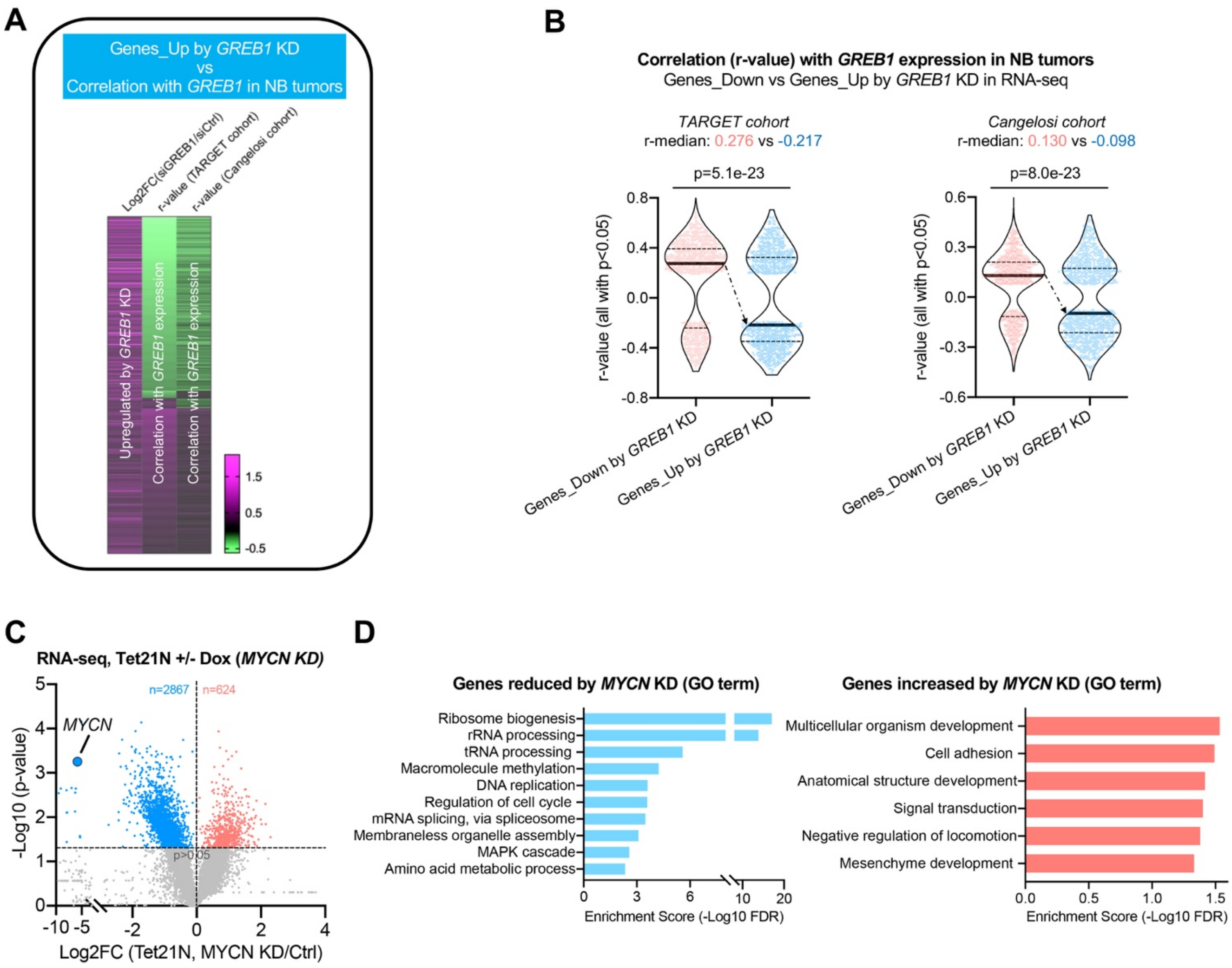

A
Genes_Up by GREB1 KD
vs
Correlation with GREB1 in NB tumors
Log2FC(siGREB1/siCtrl)
r-value (TARGET cohort)
r-value (Cangelosi cohort)
Upregulated by GREB1 KD
Correlation with GREB1 expression
Correlation with GREB1 expression
1.5
0.5
-0.5
B
Correlation (r-value) with GREB1 expression in NB tumors
Genes_Down vs Genes_Up by GREB1 KD in RNA-seq
TARGET cohort
r-median: 0.276 vs -0.217
p=5.1e-23
Cangelosi cohort
r-median: 0.130 vs -0.098
p=8.0e-23
r-value (all with p<0.05)
Genes_Down by GREB1 KD
Genes_Up by GREB1 KD
C
RNA-seq, Tet21N +/- Dox (MYCN KD)
n=2867
n=624
MYCN
p>0.05
-Log10 (p-value)
Log2FC (Tet21N, MYCN KD/Ctrl)
D
Genes reduced by MYCN KD (GO term)
Ribosome biogenesis
rRNA processing
tRNA processing
Macromolecule methylation
DNA replication
Regulation of cell cycle
mRNA splicing, via spliceosome
Membraneless organelle assembly
MAPK cascade
Amino acid metabolic process
Enrichment Score (-Log10 FDR)
Genes increased by MYCN KD (GO term)
Multicellular organism development
Cell adhesion
Anatomical structure development
Signal transduction
Negative regulation of locomotion
Mesenchyme development
Enrichment Score (-Log10 FDR)

Figure S3. Identification of a GREB1-controlled gene signature in *MNA*+ NB independent of MYCN. (A) Genes upregulated by *GREB1* KD in Kelly cells (by RNA-seq) were integrated with all genes significantly correlated with *GREB1* in both cohorts of NB tumors (based on data derived from the R2 database). **(B)** Comparison of the correlation power (r-value) with *GREB1* expression in the two cohorts of NB tumors: genes downregulated by *GREB1* KD vs genes upregulated by *GREB1* KD identified by RNA-seq in Kelly cells. **(C)** Publicly available RNA-seq analysis in Tet21N cells +/- Dox for 0h (Ctrl) or 24h (inducible MYCN KD) [ref. 41]. **(D)** Gene Ontology (GO) analysis of the gene sets significantly reduced/increased by MYCN KD in Tet21N cells. GO term analysis was performed using the PANTHER database and the GO-Slim Biological Process was analyzed. Lines in the plots in (B) represent median and quartiles, and the p values were determined by Mann-Whitney test.

Figure S4

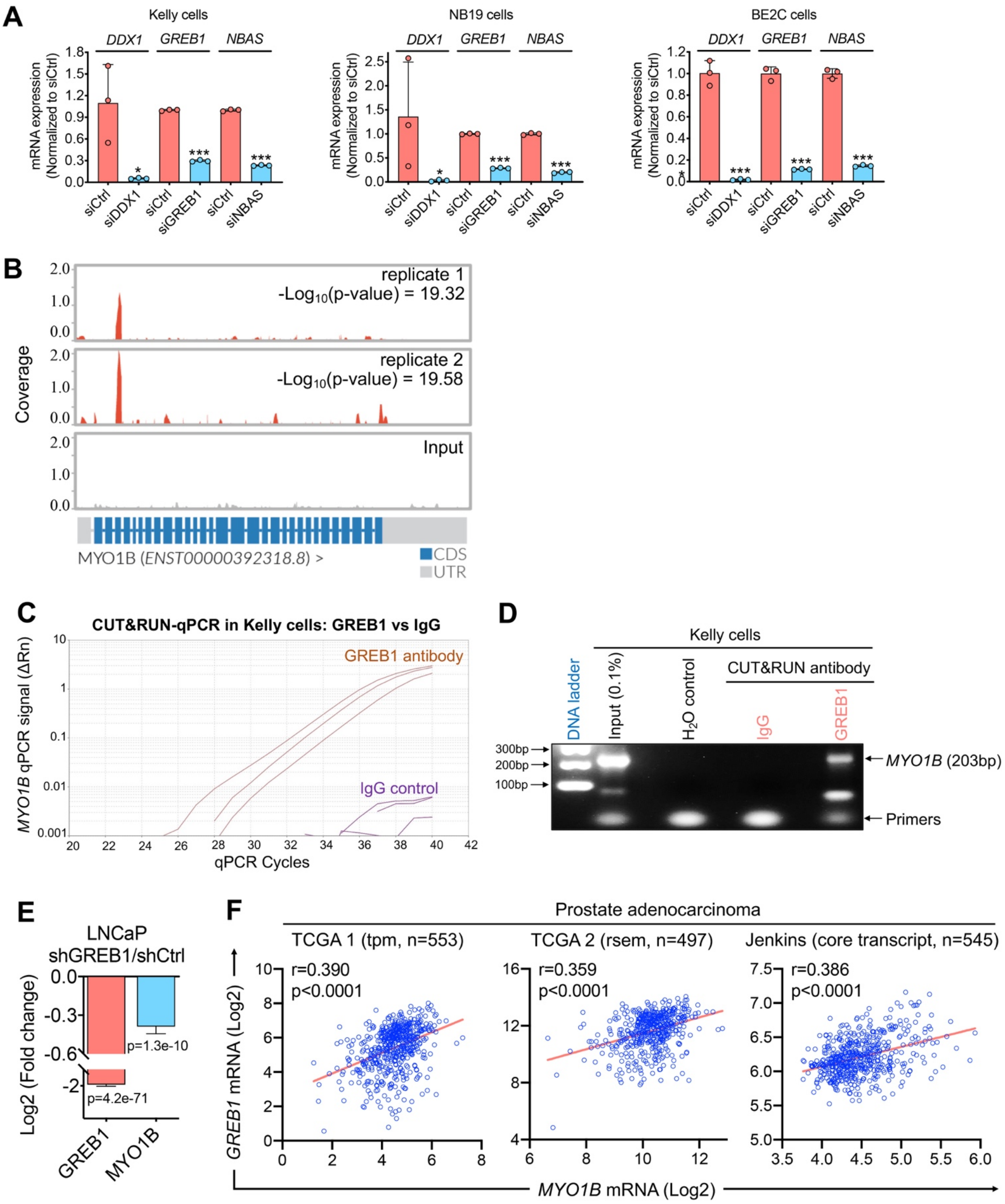

A
Kelly cells
NB19 cells
BE2C cells
DDX1
GREB1
NBAS
mRNA expression (Normalized to siCtrl)
siCtrl
siDDX1
siGREB1
siNBAS
B
replicate 1
-Log10(p-value) = 19.32
replicate 2
-Log10(p-value) = 19.58
Input
Coverage
MYO1B (ENST00000392318.8) >
CDS
UTR
C
CUT&RUN-qPCR in Kelly cells: GREB1 vs IgG
GREB1 antibody
IgG control
MYO1B qPCR signal (ΔRn)
qPCR Cycles
D
Kelly cells
CUT&RUN antibody
DNA ladder
Input (0.1%)
H2O control
IgG
GREB1
300bp
200bp
100bp
MYO1B (203bp)
Primers
E
LNCaP
shGREB1/shCtrl
Log2 (Fold change)
p=1.3e-10
p=4.2e-71
GREB1
MYO1B
F
Prostate adenocarcinoma
TCGA 1 (tpm, n=553)
TCGA 2 (rsem, n=497)
Jenkins (core transcript, n=545)
r=0.390
p<0.0001
r=0.359
p<0.0001
r=0.386
p<0.0001
GREB1 mRNA (Log2)
MYO1B mRNA (Log2)

Figure S4. Identification of *MYO1B* as a GREB-controlled gene. (A) Gene expression changes upon knockdown of the indicated genes were assessed by qPCR (n=3). **(B)** GREB1 binding at the *MYO1B* locus uncovered by GREB1 ChIP-seq in MCF7 cells (data were derived from GSE41561 deposited in Gene Expression Omnibus). **(C-D)** Binding of GREB1 to the *MYO1B* locus at Exon 3, as evidenced by GREB1 ChIP-seq data shown in (B), was validated by GREB1 CUT&RUN analysis (i.e. Cleavage Under Targets and Release Using Nuclease, an alternative approach for *in situ* ChIP analysis[44]) in Kelly cells. **(E)** Gene expression changes of *GREB1* and *MYO1B* upon stable *GREB1* knockdown in prostate adenocarcinoma LNCaP cells. The data was derived from RNA-seq analysis deposited in Gene Expression Omnibus GSE120720. **(F)** Correlation between *GREB1* and *MYO1B* mRNA expression in three cohorts of prostate adenocarcinoma samples based on data extracted from the R2 database. For all panels, data presented are means ± SD; p values were determined by two-tailed unpaired Student's t-test, $*p < 0.05$, $***p < 0.001$. Pearson coefficient analysis was performed to determine correlations between two variables.

Figure S5

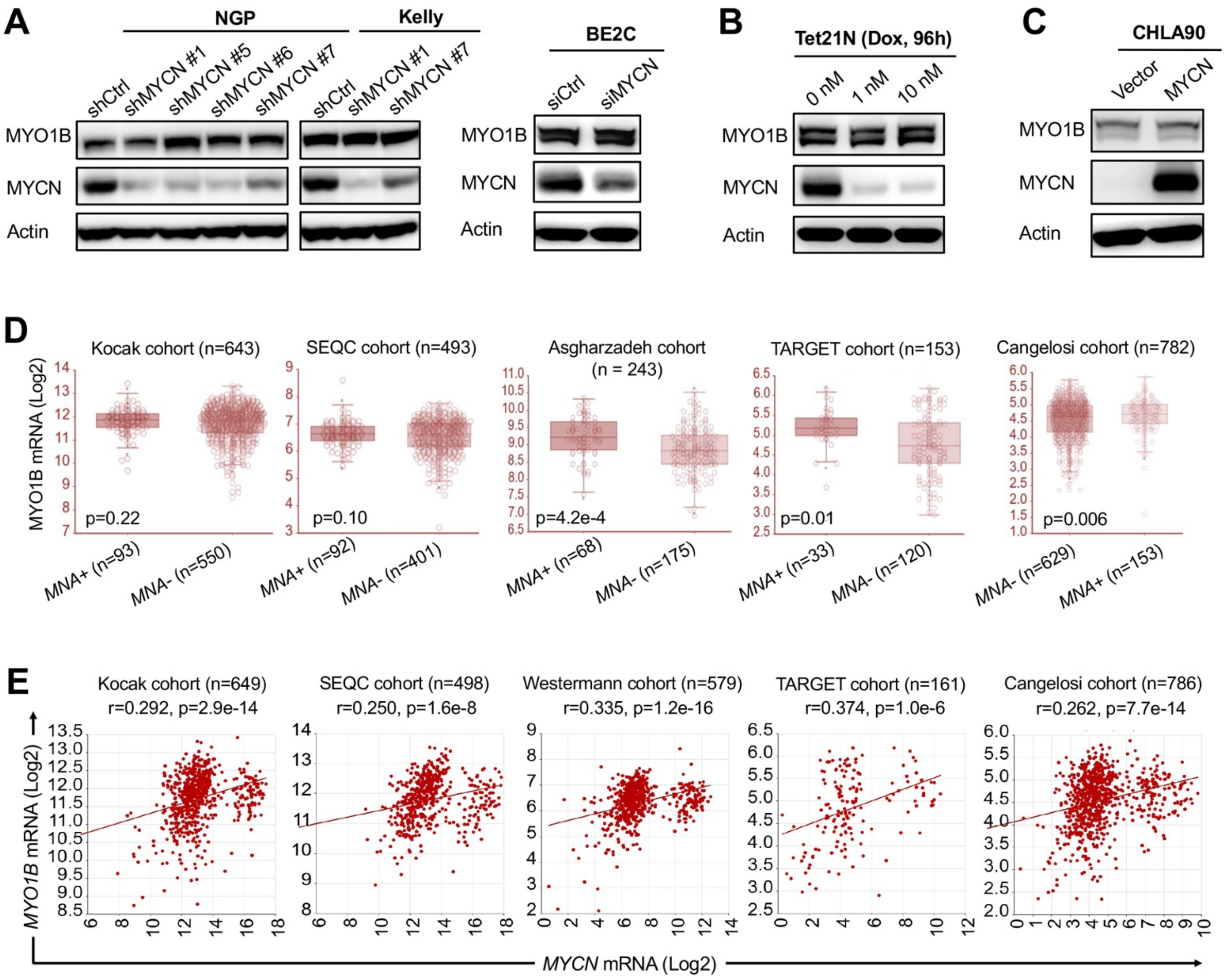

A
NGP
Kelly
shCtrl
shMYCN #1
shMYCN #5
shMYCN #6
shMYCN #7
MYO1B
MYCN
Actin
BE2C
siCtrl
siMYCN
B
Tet21N (Dox, 96h)
0 nM
1 nM
10 nM
C
CHLA90
Vector
MYCN
D
Kocak cohort (n=643)
SEQC cohort (n=493)
Asgharzadeh cohort (n = 243)
TARGET cohort (n=153)
Cangelosi cohort (n=782)
MYO1B mRNA (Log2)
p=0.22
p=0.10
p=4.2e-4
p=0.01
p=0.006
MNA+ (n=93)
MNA- (n=550)
MNA+ (n=92)
MNA- (n=401)
MNA+ (n=68)
MNA- (n=175)
MNA+ (n=33)
MNA- (n=120)
MNA- (n=629)
MNA+ (n=153)
E
Kocak cohort (n=649)
r=0.292, p=2.9e-14
SEQC cohort (n=498)
r=0.250, p=1.6e-8
Westermann cohort (n=579)
r=0.335, p=1.2e-16
TARGET cohort (n=161)
r=0.374, p=1.0e-6
Cangelosi cohort (n=786)
r=0.262, p=7.7e-14
MYO1B mRNA (Log2)
MYCN mRNA (Log2)

Figure S5. MYCN does not regulate *MYO1B* expression in neuroblastoma. (A-C) Protein expression changes upon *MYCN* knockdown or overexpression in a panel of NB cell lines were assessed by Immunoblotting. **(D)** Comparison of *MYO1B* mRNA expression levels in *MNA*- vs *MNA+* NB samples in five patient cohorts using data extracted from the R2 database. **(E)** Correlation between *MYO1B* and *MYCN* mRNA expression in the five NB patient cohorts shown in (D). Statistics and p-values in all panels were provided by the R2 database.

Figure S6,

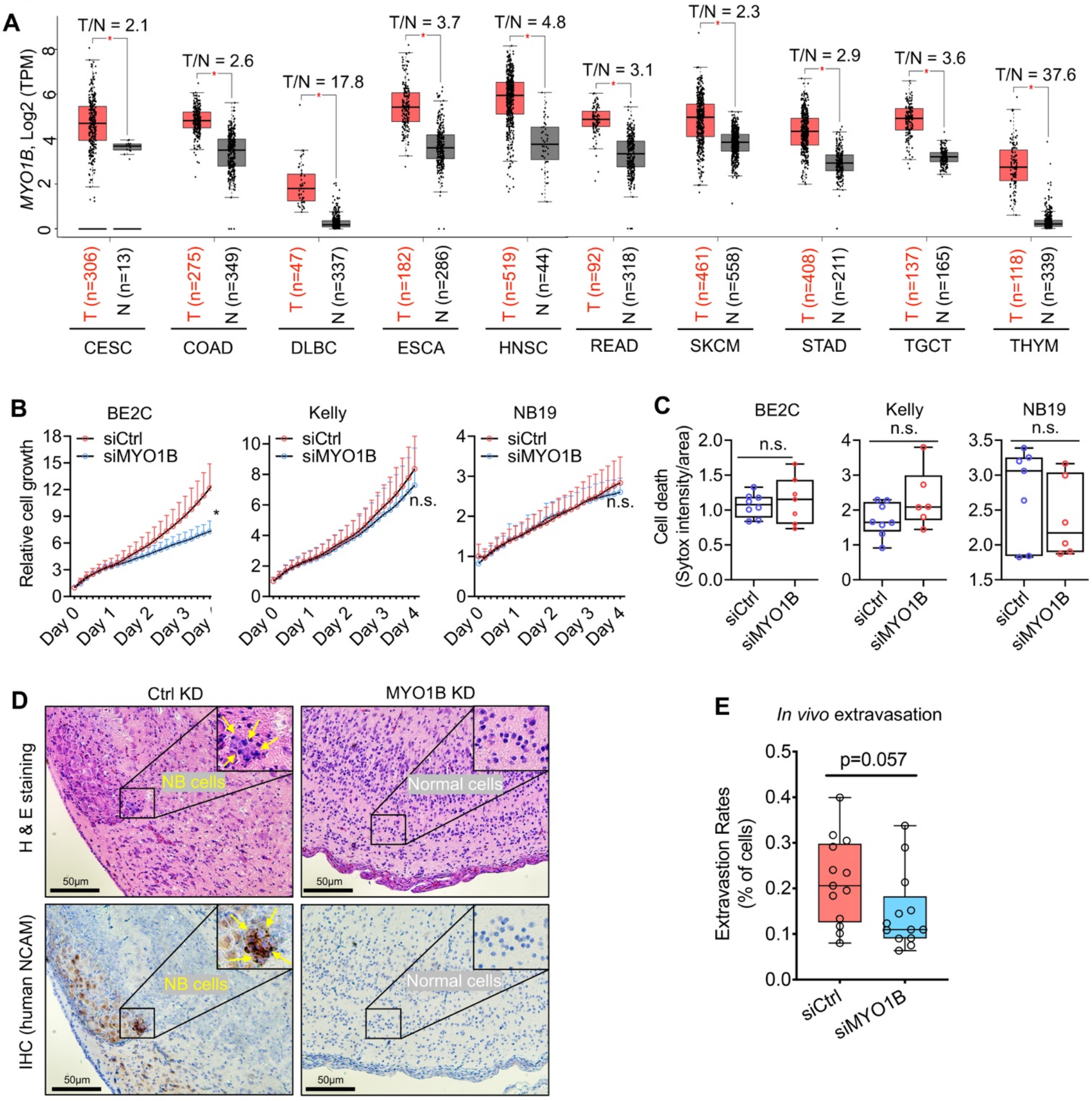

A
MYO1B, Log2 (TPM)
T/N = 2.1
T/N = 2.6
T/N = 17.8
T/N = 3.7
T/N = 4.8
T/N = 3.1
T/N = 2.3
T/N = 2.9
T/N = 3.6
T/N = 37.6
T (n=306) N (n=13)
CESC
T (n=275) N (n=349)
COAD
T (n=47) N (n=337)
DLBC
T (n=182) N (n=286)
ESCA
T (n=519) N (n=44)
HNSC
T (n=92) N (n=318)
READ
T (n=461) N (n=558)
SKCM
T (n=408) N (n=211)
STAD
T (n=137) N (n=165)
TGCT
T (n=118) N (n=339)
THYM
B
BE2C
Kelly
NB19
Relative cell growth
siCtrl
siMYO1B
n.s.
Day 0
Day 1
Day 2
Day 3
Day 4
C
Cell death (Sytox intensity/area)
n.s.
siCtrl
siMYO1B
D
Ctrl KD
MYO1B KD
H & E staining
IHC (human NCAM)
NB cells
Normal cells
50µm
E
In vivo extravasation
Extravasation Rates (% of cells)
p=0.057
siCtrl
siMYO1B

Figure S6. Marked upregulation of MYO1B in human cancers and its biological function in NB. **(A)** Comparison of *MYO1B* mRNA expression levels in normal (N) and tumor (T) samples across ten human cancer types. The plots were directly generated using tools within the GEPIA (Gene Expression Profiling Interactive Analysis) database. **(B-C)** The impact of MYO1B depletion on NB cell growth (A) and cell death (Sytox Orange staining) were measured by Incycyte. **(D)** Identification of metastatic human NB nodules by H&E staining and human NCAM staining by IHC in serial tissue sections. **(E)** The impact of MYO1B depletion on NB cell (Kelly) extravasation was evaluated using the chick embryo chorioallantoic membrane (CAM) metastasis model. Difference between groups were determined by two-tailed unpaired Student's t-test, n.s., not significant, $^*p < 0.05$.

Figure S7

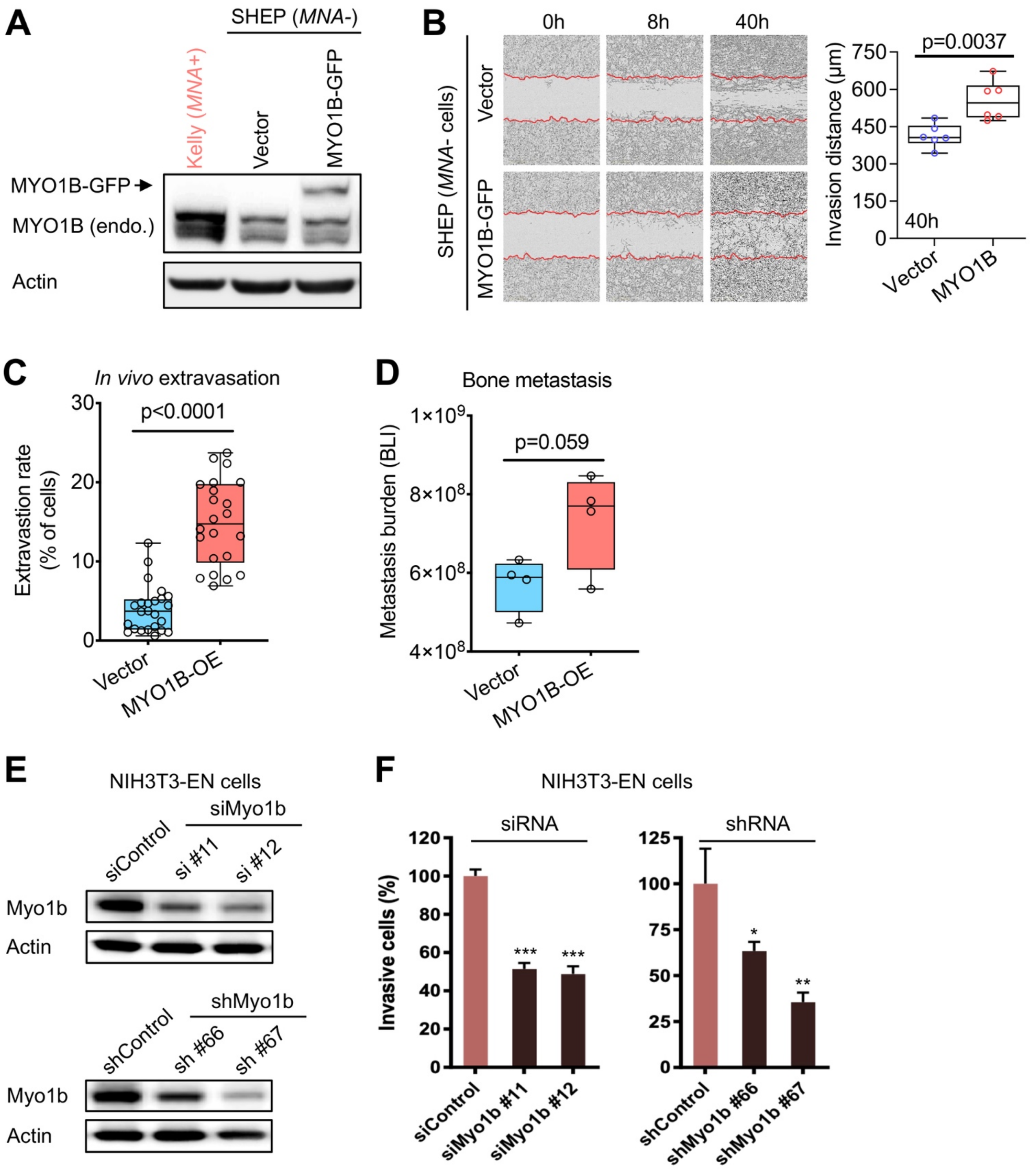
A
SHEP (MNA-)
Kelly (MNA+)
Vector
MYO1B-GFP
MYO1B-GFP
MYO1B (endo.)
Actin
B
0h
8h
40h
SHEP (MNA- cells)
Vector
MYO1B-GFP
p=0.0037
Invasion distance (μm)
40h
Vector
MYO1B
C
In vivo extravasation
p<0.0001
Extravasation rate (% of cells)
Vector
MYO1B-OE
D
Bone metastasis
p=0.059
Metastasis burden (BLI)
Vector
MYO1B-OE
E
NIH3T3-EN cells
siMyo1b
siControl
si #11
si #12
Myo1b
Actin
shMyo1b
shControl
sh #66
sh #67
Myo1b
Actin
F
NIH3T3-EN cells
siRNA
shRNA
Invasive cells (%)
siControl
siMyo1b #11
siMyo1b #12
shControl
shMyo1b #66
shMyo1b #67

Figure S7. The role of MYO1B in the invasiveness and metastatic capacity of *MNA*- tumor cells. **(A)** MYO1B-GFP overexpression (OE) in SHEP cells was validated by Western blotting, Kelly cells were used as a MYO1B positive control. **(B)** The impact of MYO1B OE on cell invasion through matrigel was evaluated using Essen BioScience Incucyte 96-well scratch wound invasion assay (n=6). **(C-D)** The impact of MYO1B OE on the extravasation and metastatic capacity of SHEP cells (luciferase-expressing) was evaluated using the chick embryo chorioallantoic membrane (CAM) metastasis model. Right upper-panel, metastasis burden was measured by bioluminescent intensity (BLI) in each embryo; right lower-panel, BLI over 5x10e5 was regarded as high metastasis burden. **(E-F)** The impact of Myo1b depletion (E) on NIH3T3-EN cell invasiveness through Matrigel was determined by Essen BioScience Incucyte 96-well scratch wound invasion assay (n=6). Difference between groups were determined by two-tailed unpaired Student's t-test, *$p < 0.05$, **$p < 0.01$, ***$p < 0.001$.

Figure S8

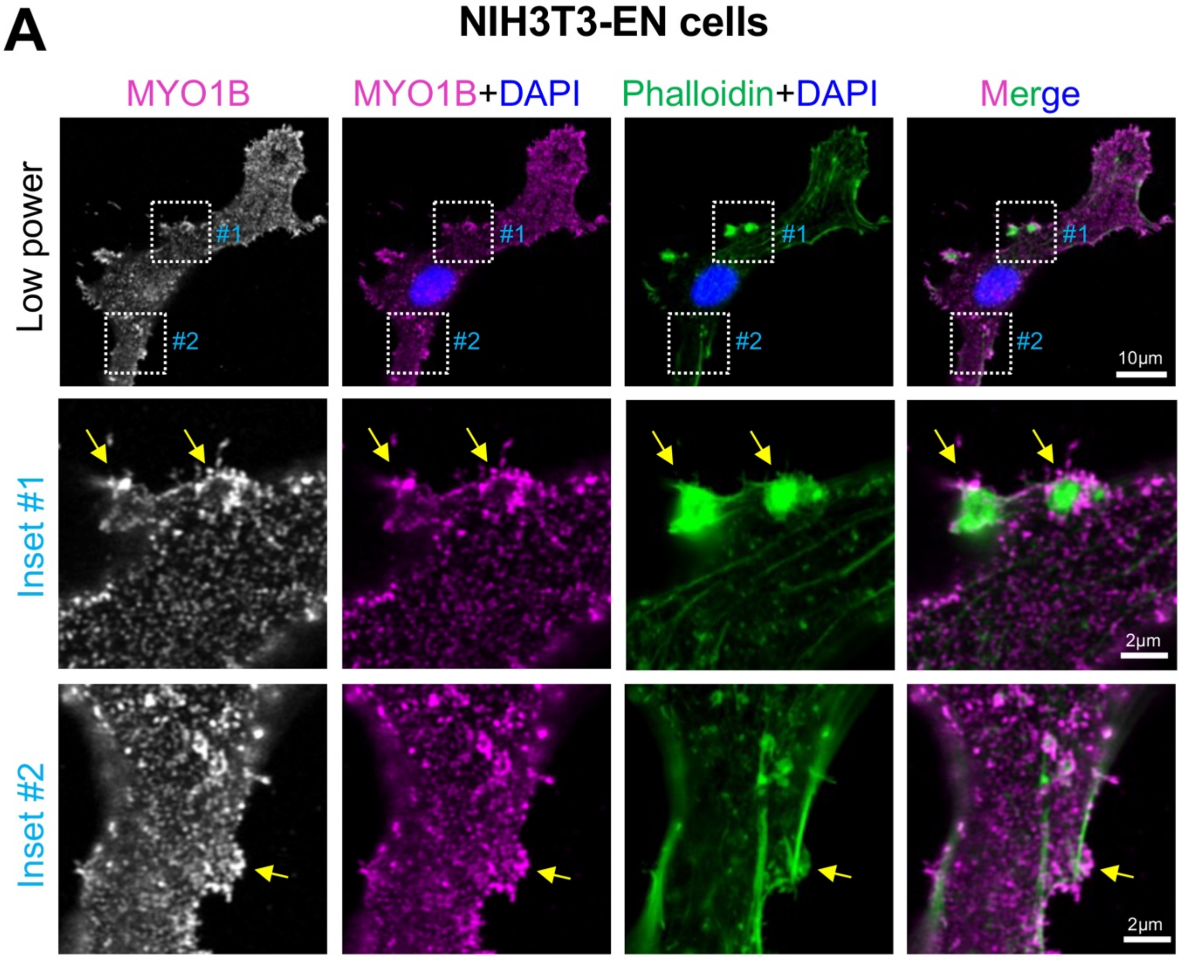
A
NIH3T3-EN cells
MYO1B
MYO1B+DAPI
Phalloidin+DAPI
Merge
Low power
Inset #1
Inset #2
#1
#2
10μm
2μm
2μm

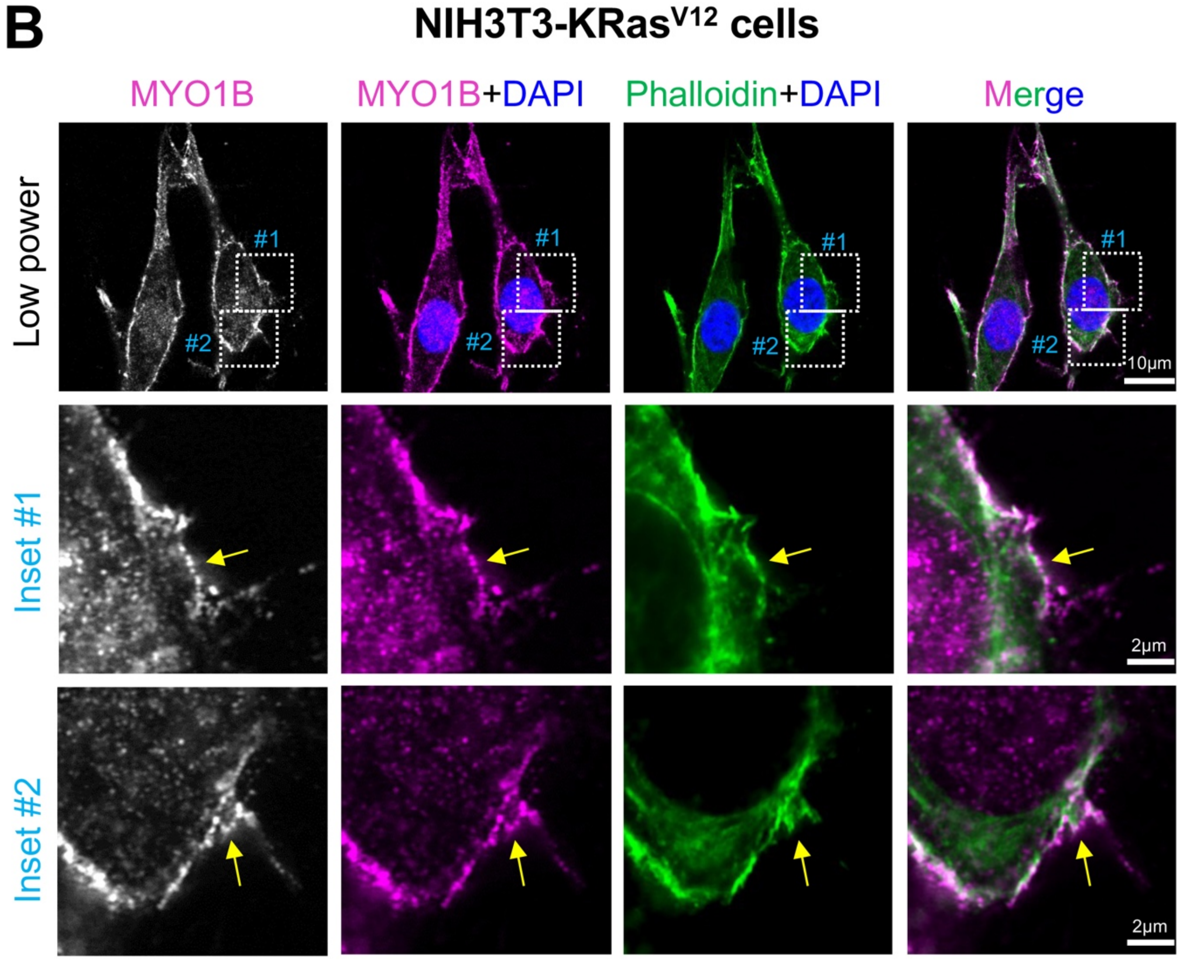
B
NIH3T3-KRas^{V12} cells
MYO1B
MYO1B+DAPI
Phalloidin+DAPI
Merge
Low power
Inset #1
Inset #2
#1
#2
10μm
2μm
2μm

Figure S8. MYO1B co-localizes with budding structures on the cell surface of transformed cells. (A-B) Co-localization of MYO1B with cytoskeletal structures on the cell surface of NIH3T3 cells transformed by oncogenic ETV6-NTRK3 (A) or mutant $KRas^{V12}$ (B) was evaluated by immunofluorescence confocal microscopy.

Figure S9

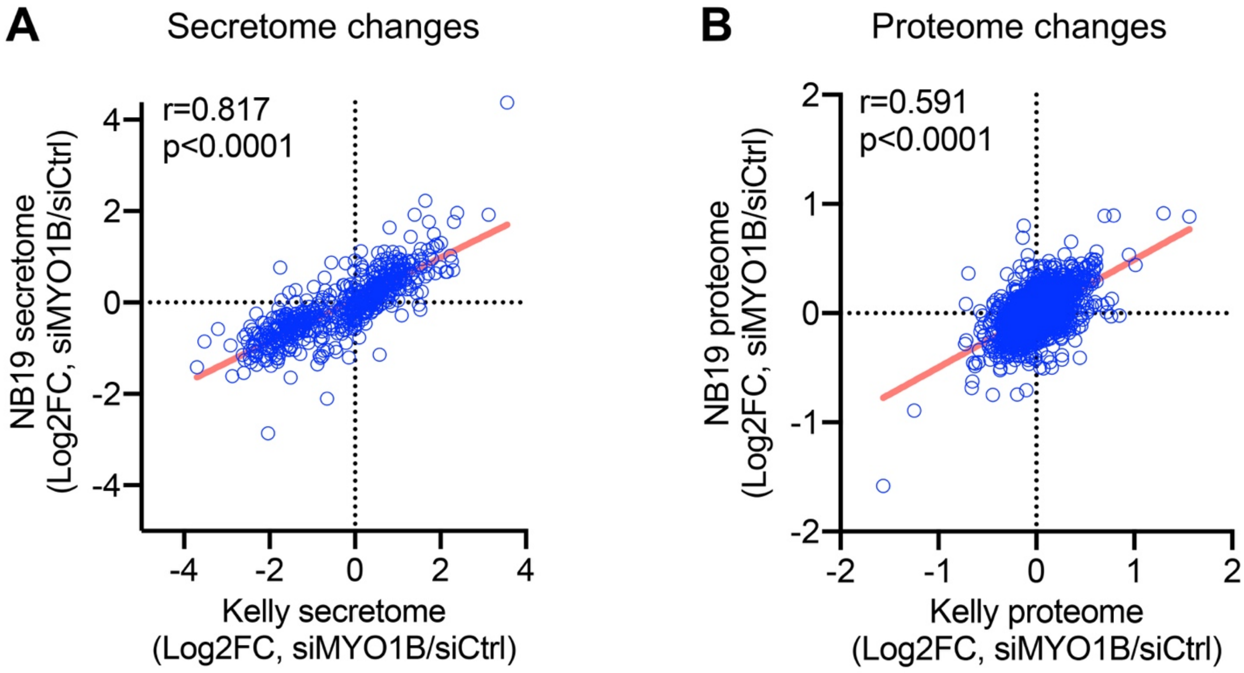
A
Secretome changes
r=0.817
p<0.0001
NB19 secretome
(Log2FC, siMYO1B/siCtrl)
Kelly secretome
(Log2FC, siMYO1B/siCtrl)
4
2
0
-2
-4
-4
-2
0
2
4
B
Proteome changes
r=0.591
p<0.0001
NB19 proteome
(Log2FC, siMYO1B/siCtrl)
Kelly proteome
(Log2FC, siMYO1B/siCtrl)
2
1
0
-1
-2
-2
-1
0
1
2

Figure S9. Concordant changes in our secretome and proteome analysis between two NB cell lines. **(A-B)** Statistical analysis of the concordant secretome changes (A) and proteome changes (B) in Kelly and NB19 cells. Pearson coefficient analysis was performed to determine correlations between two variables.

Figure S10

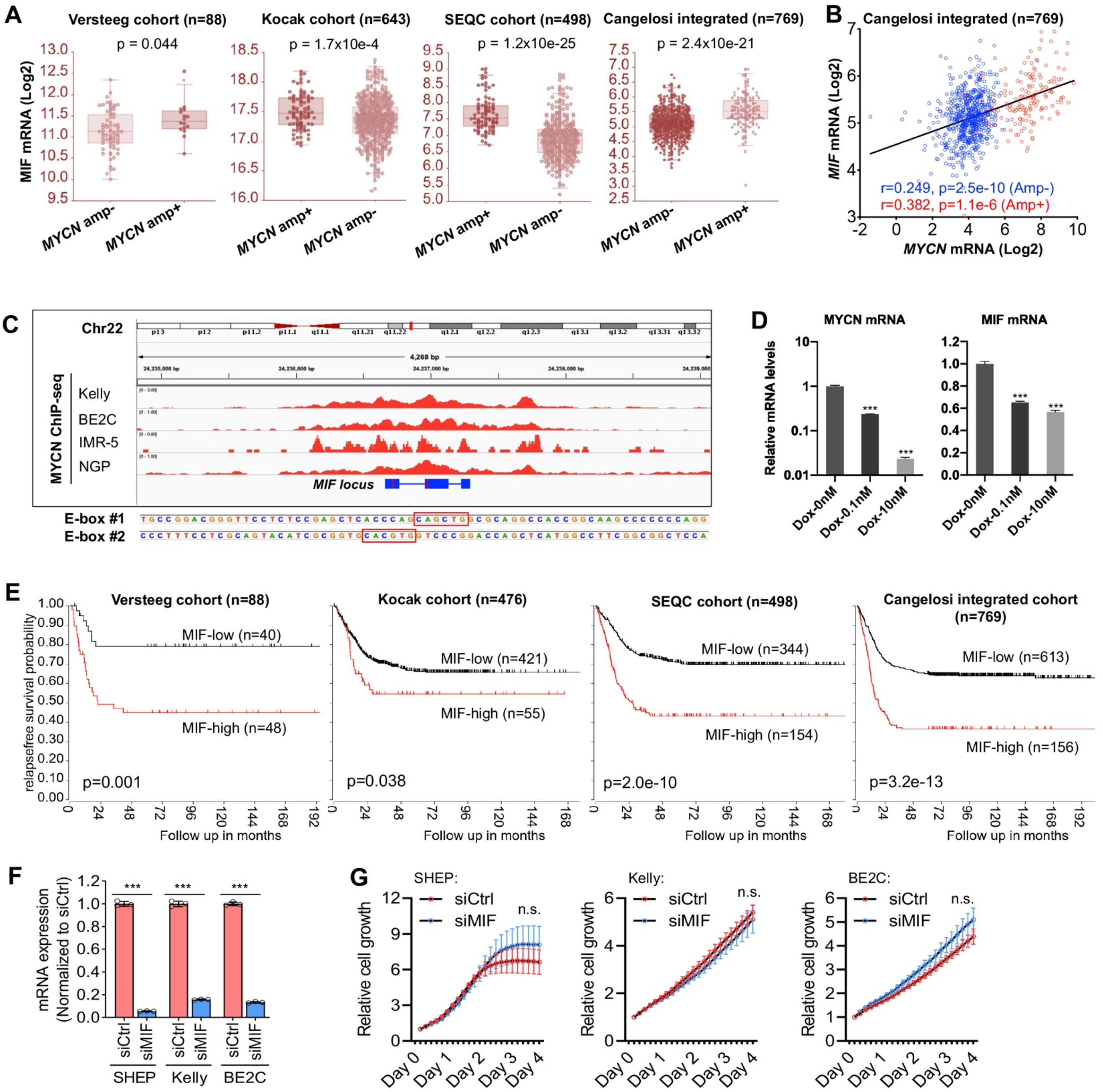
A
Versteeg cohort (n=88)
p = 0.044
Kocak cohort (n=643)
p = 1.7x10e-4
SEQC cohort (n=498)
p = 1.2x10e-25
Cangelosi integrated (n=769)
p = 2.4x10e-21
MIF mRNA (Log2)
MYCN amp-
MYCN amp+
B
Cangelosi integrated (n=769)
r=0.249, p=2.5e-10 (Amp-)
r=0.382, p=1.1e-6 (Amp+)
MIF mRNA (Log2)
MYCN mRNA (Log2)
C
Chr22
MYCN ChIP-seq
Kelly
BE2C
IMR-5
NGP
MIF locus
E-box #1
E-box #2
D
MYCN mRNA
MIF mRNA
Relative mRNA leivels
Dox-0nM
Dox-0.1nM
Dox-10nM
E
Versteeg cohort (n=88)
MIF-low (n=40)
MIF-high (n=48)
p=0.001
Kocak cohort (n=476)
MIF-low (n=421)
MIF-high (n=55)
p=0.038
SEQC cohort (n=498)
MIF-low (n=344)
MIF-high (n=154)
p=2.0e-10
Cangelosi integrated cohort (n=769)
MIF-low (n=613)
MIF-high (n=156)
p=3.2e-13
relapsefree survival probability
Follow up in months
F
mRNA expression (Normalized to siCtrl)
siCtrl
siMIF
SHEP
Kelly
BE2C
G
SHEP:
Kelly:
BE2C:
siCtrl
siMIF
n.s.
Relative cell growth
Day 0
Day 1
Day 2
Day 3
Day 4

Figure S10. MIF regulated by MYCN is highly expressed in MNA+ NB and promotes aggressiveness and predicts poor prognosis. (A) Comparison of *MIF* mRNA expression levels in *MNA*- vs *MNA+* NB samples in distinct patient cohorts using data extracted from the R2 database. **(B)** Correlation between *MIF* and *MYCN* mRNA expression NB patient samples in a dataset extracted from the R2 database. **(C)** MYCN binding at the *MIF* locus in various *MNA+* NB cell lines as revealed by MYCN ChIP-seq extracted from the ChIP Atlas database. **(D)** qPCR analysis of *MYCN* and *MIF* changes at 96h upon MYCN depletion induced by doxycycline (Dox) in Tet21N cells, a NB cell line that expresses *MYCN* cDNA under the control of a Tet-off system. **(E)** The prognostic significance of *MIF* mRNA expression in various cohorts of NB patients. Data were extracted from the R2 database. **(F-G)** The impact of *MIF* depletion (F) on NB cell growth was evaluated by Incucyte (n=6-8). Difference between groups were determined by two-tailed unpaired Student's t-test, ***$p < 0.001$. Pearson coefficient analysis was performed to determine correlations between two variables. Log-rank test was used in Kaplan-Meier survival analysis.

Figure S11

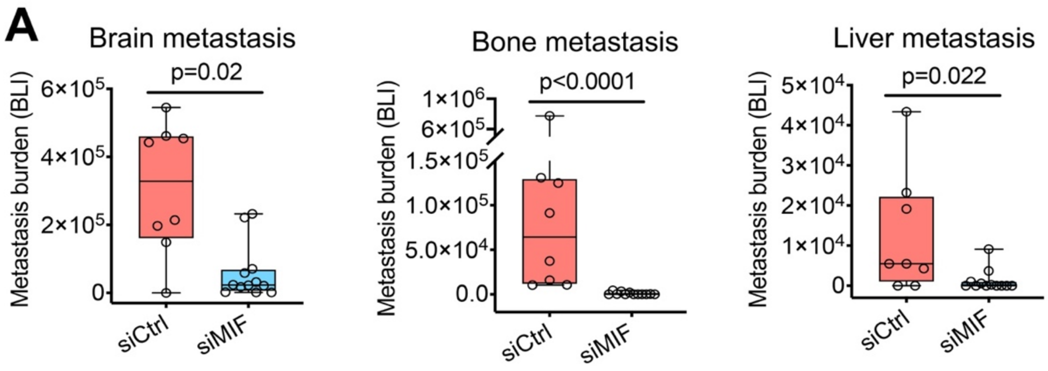
A
Brain metastasis
p=0.02
Metastasis burden (BLI)
6×10^5
4×10^5
2×10^5
0
siCtrl
siMIF
Bone metastasis
p<0.0001
1×10^6
6×10^5
1.5×10^5
1.0×10^5
5.0×10^4
0.0
Liver metastasis
p=0.022
5×10^4
4×10^4
3×10^4
2×10^4
1×10^4

Figure S11. (A) The impact of *MIF* depletion on the metastatic capacity of NB cells (luciferase-expressing Kelly cells) was evaluated using the chick embryo chorioallantoic membrane (CAM) metastasis model (n=8-12), and the metastasis burden was measured by bioluminescent intensity (BLI).

Figure S12

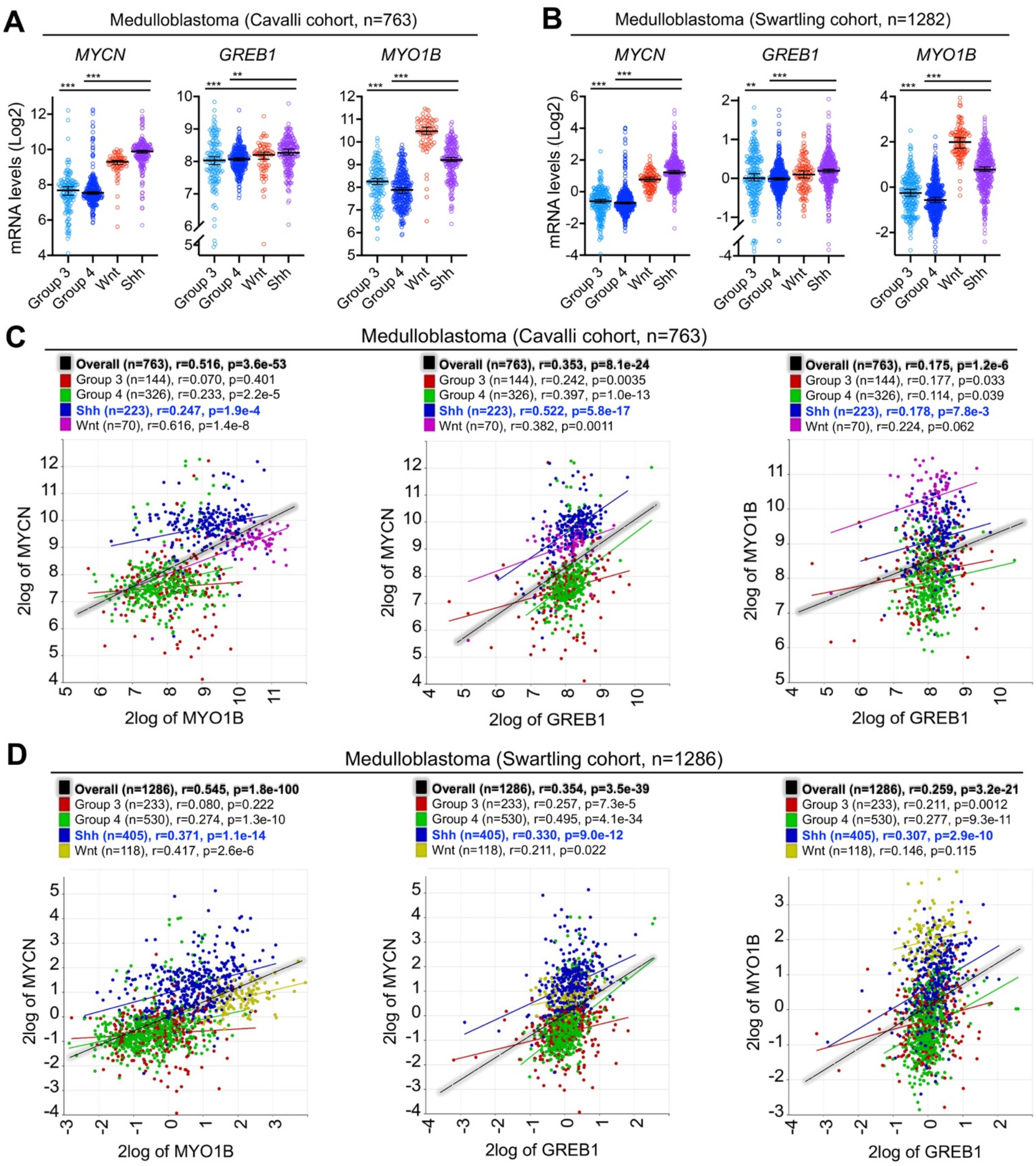
A
Medulloblastoma (Cavalli cohort, n=763)
MYCN
GREB1
MYO1B
mRNA levels (Log2)
Group 3
Group 4
Wnt
Shh
B
Medulloblastoma (Swartling cohort, n=1282)
C
Medulloblastoma (Cavalli cohort, n=763)
Overall (n=763), r=0.516, p=3.6e-53
Group 3 (n=144), r=0.070, p=0.401
Group 4 (n=326), r=0.233, p=2.2e-5
Shh (n=223), r=0.247, p=1.9e-4
Wnt (n=70), r=0.616, p=1.4e-8
2log of MYCN
2log of MYO1B
Overall (n=763), r=0.353, p=8.1e-24
Group 3 (n=144), r=0.242, p=0.0035
Group 4 (n=326), r=0.397, p=1.0e-13
Shh (n=223), r=0.522, p=5.8e-17
Wnt (n=70), r=0.382, p=0.0011
2log of GREB1
Overall (n=763), r=0.175, p=1.2e-6
Group 3 (n=144), r=0.177, p=0.033
Group 4 (n=326), r=0.114, p=0.039
Shh (n=223), r=0.178, p=7.8e-3
Wnt (n=70), r=0.224, p=0.062
2log of MYO1B
2log of GREB1
D
Medulloblastoma (Swartling cohort, n=1286)
Overall (n=1286), r=0.545, p=1.8e-100
Group 3 (n=233), r=0.080, p=0.222
Group 4 (n=530), r=0.274, p=1.3e-10
Shh (n=405), r=0.371, p=1.1e-14
Wnt (n=118), r=0.417, p=2.6e-6
2log of MYCN
2log of MYO1B
Overall (n=1286), r=0.354, p=3.5e-39
Group 3 (n=233), r=0.257, p=7.3e-5
Group 4 (n=530), r=0.495, p=4.1e-34
Shh (n=405), r=0.330, p=9.0e-12
Wnt (n=118), r=0.211, p=0.022
2log of GREB1
Overall (n=1286), r=0.259, p=3.2e-21
Group 3 (n=233), r=0.211, p=0.0012
Group 4 (n=530), r=0.277, p=9.3e-11
Shh (n=405), r=0.307, p=2.9e-10
Wnt (n=118), r=0.146, p=0.115
2log of MYO1B
2log of GREB1

Figure S12. MYO1B is overexpressed in *MNA*+ medulloblastoma. **(A-B)** Comparison of the indicated gene expression levels across the four major subgroups of medulloblastoma from the Cavalli cohort (A) and the Swartling cohort (B). The plots were generated from data extracted from the R2 database. **(C-D)** Correlation among the mRNA expression of *MYCN, MYO1B and GREB1* across the four major subgroups of medulloblastoma from the Cavalli cohort (C) and the Swartling cohort (D). The plots were directly generated using tools within the R2 database. Difference between groups were determined by two-tailed unpaired Student's t-test, **p < 0.01, ***p < 0.001. Pearson coefficient analysis was performed to determine correlations between two variables.

Supplementary Table S1-S14 (Separate files)

Table S1. RNA-seq in Kelly cells +/- GREB1 KD.

Table S2. Genes significantly correlated with GREB1 expression in the TARGET cohort (n=161).

Table S3. Genes significantly correlated with GREB1 expression in the Cangelosi cohort (n=786).

Table S4. The 583-gene list shown in Fig. 2C-i.

Table S5. Genes significantly correlated with MYCN expression in the TARGET cohort (n=161).

Table S6. Genes significantly correlated with MYCN expression in the Cangelosi cohort (n=786).

Table S7. The 388 GREB1-signature genes in MNA+ NB.

Table S8. RNA-seq in Tet21N cells +/- MYCN depletion, public dataset GSE80153.

Table S9. The 658 MYCN-signature genes in MNA+ NB.

Table S10. NB19_secretome, siMYO1B/siCtrl.

Table S11. Kelly_secretome, siMYO1B/siCtrl.

Table S12. NB19_total proteome, siMYO1B/siCtrl.

Table S13. Kelly_total proteome, siMYO1B/siCtrl.

Table S14. Cytokine and growth factors in the secretome significantly reduced by MYO1B depletion.

REFERENCES AND NOTES

1. J. M. Maris, Recent advances in neuroblastoma. *N. Engl. J. Med.* **362**, 2202–2211 (2010).

2. C. U. Louis, J. M. Shohet, Neuroblastoma: Molecular pathogenesis and therapy. *Annu. Rev. Med.* **66**, 49–63 (2015).

3. K. K. Matthay, J. M. Maris, G. Schleiermacher, A. Nakagawara, C. L. Mackall, L. Diller, W. A. Weiss, Neuroblastoma. *Nat. Rev. Dis. Primers* **2**, 16078 (2016).

4. G. M. Brodeur, R. C. Seeger, M. Schwab, H. E. Varmus, J. M. Bishop, Amplification of N-myc in untreated human neuroblastomas correlates with advanced disease stage. *Science* **224**, 1121–1124 (1984).

5. S. L. Cohn, A. D. J. Pearson, W. B. London, T. Monclair, P. F. Ambros, G. M. Brodeur, A. Faldum, B. Hero, T. Iehara, D. Machin, V. Mosseri, T. Simon, A. Garaventa, V. Castel, K. K. Matthay; INRG Task Force, The International Neuroblastoma Risk Group (INRG) classification system: An INRG Task Force report. *J. Clin. Oncol.* **27**, 289–297 (2009).

6. M. Huang, W. A. Weiss, Neuroblastoma and MYCN. *Cold Spring Harb. Perspect. Med.* **3**, a014415 (2013).

7. J. T. Powers, K. M. Tsanov, D. S. Pearson, F. Roels, C. S. Spina, R. Ebright, M. Seligson, Y. de Soysa, P. Cahan, J. Theißen, H. C. Tu, A. Han, K. C. Kurek, G. S. LaPier, J. K. Osborne, S. J. Ross, M. Cesana, J. J. Collins, F. Berthold, G. Q. Daley, Multiple mechanisms disrupt the let-7 microRNA family in neuroblastoma. *Nature* **535**, 246–251 (2016).

8. J. A. Squire, P. S. Thorner, S. Weitzman, J. D. Maggi, P. Dirks, J. Doyle, M. Hale, R. Godbout, Co-amplification of MYCN and a DEAD box gene (DDX1) in primary neuroblastoma. *Oncogene* **10**, 1417–1422 (1995).

9. C. F. Manohar, H. R. Salwen, G. M. Brodeur, S. L. Cohn, Co-amplification and concomitant high levels of expression of a DEAD box gene with MYCN in human neuroblastoma. *Genes Chromosomes Cancer* **14**, 196–203 (1995).

10. A. Pandita, J. Bayani, J. Paderova, P. Marrano, C. Graham, M. Barrett, M. Prasad, M. Zielenska, J. A. Squire, Integrated cytogenetic and high-resolution array CGH analysis of genomic alterations associated with MYCN amplification. *Cytogenet. Genome Res.* **134**, 27–39 (2011).

11. P. Depuydt, V. Boeva, T. D. Hocking, R. Cannoodt, I. M. Ambros, P. F. Ambros, S. Asgharzadeh, E. F. Attiyeh, V. Combaret, R. Defferrari, M. Fischer, B. Hero, M. D. Hogarty, M. S. Irwin, J. Koster, S. Kreissman, R. Ladenstein, E. Lapouble, G. Laureys, W. B. London, K. Mazzocco, A. Nakagawara, R. Noguera, M. Ohira, J. R. Park, U. Pötschger, J. Theissen, G. P. Tonini, D. Valteau-Couanet, L. Varesio, R. Versteeg, F. Speleman, J. M. Maris, G. Schleiermacher, K. De Preter, Genomic amplifications and distal 6q loss: Novel markers for poor survival in high-risk neuroblastoma patients. *J. Natl. Cancer Inst.* **110**, 1084–1093 (2018).

12. K. Helmsauer, M. E. Valieva, S. Ali, R. C. González, R. Schöpflin, C. Röefzaad, Y. Bei, H. D. Garcia, E. Rodriguez-Fos, M. Puiggròs, K. Kasack, K. Haase, C. Keskeny, C. Y. Chen, L. P. Kuschel, P. Euskirchen, V. Heinrich, M. I. Robson, C. Rosswog, J. Toedling, A. Szymansky, F. Hertwig, M. Fischer, D. Torrents, A. Eggert, J. H. Schulte, S. Mundlos, A. G. Henssen, R. P. Koche, Enhancer hijacking determines extrachromosomal circular MYCN amplicon architecture in neuroblastoma. *Nat. Commun.* **11**, 5823 (2020).

13. H. Mohammed, C. D'Santos, A. A. Serandour, H Raza Ali, G. D. Brown, A. Atkins, O. M. Rueda, K. A. Holmes, V. Theodorou, J. L. L. Robinson, W. Zwart, A. Saadi, C. S. Ross-Innes, S.-F. Chin, S. Menon, J. Stingl, C. Palmieri, C. Caldas, J. S. Carroll, Endogenous purification reveals GREB1 as a key estrogen receptor regulatory factor. *Cell Rep.* **3**, 342–349 (2013).

14. E. M. Shin, V. T. Huynh, S. A. Neja, C. Y. Liu, A. Raju, K. Tan, N. S. Tan, J. Gunaratne, X. Bi, L. M. Iyer, L. Aravind, V. Tergaonkar, GREB1: An evolutionarily conserved protein with a glycosyltransferase domain links ERα glycosylation and stability to cancer. *Sci. Adv.* **7**, eabe2470 (2021).

15. J. M. Rae, M. D. Johnson, K. E. Cordero, J. O. Scheys, J. M. Larios, M. M. Gottardis, K. J. Pienta, M. E. Lippman, GREB1 is a novel androgen-regulated gene required for prostate cancer growth. *Prostate* **66**, 886–894 (2006).

16. M. Cheng, S. Michalski, R. Kommagani, Role for growth regulation by estrogen in breast cancer 1 (GREB1) in hormone-dependent cancers. *Int. J. Mol. Sci.* **19**, 2543 (2018).

17. K. Hodgkinson, L. A. Forrest, N. Vuong, K. Garson, B. Djordjevic, B. C. Vanderhyden, GREB1 is an estrogen receptor-regulated tumour promoter that is frequently expressed in ovarian cancer. *Oncogene* **37**, 5873–5886 (2018).

18. E. Lee, J. Wongvipat, D. Choi, P. Wang, Y. S. Lee, D. Zheng, P. A. Watson, A. Gopalan, C. L. Sawyers, *GREB1* amplifies androgen receptor output in human prostate cancer and contributes to antiandrogen resistance. *eLife* **8**, e41913 (2019).

19. S. Matsumoto, T. Yamamichi, K. Shinzawa, Y. Kasahara, S. Nojima, T. Kodama, S. Obika, T. Takehara, E. Morii, H. Okuyama, A. Kikuchi, GREB1 induced by Wnt signaling promotes development of hepatoblastoma by suppressing TGFβ signaling. *Nat. Commun.* **10**, 3882 (2019).

20. M. Brunetti, I. Panagopoulos, L. Gorunova, B. Davidson, S. Heim, F. Micci, RNA-sequencing identifies novel GREB1-NCOA2 fusion gene in a uterine sarcoma with the chromosomal translocation t(2;8)(p25;q13). *Genes Chromosomes Cancer* **57**, 176–181 (2018).

21. C. H. Lee, Y. C. Kao, W. R. Lee, Y. W. Hsiao, T. P. Lu, C. Y. Chu, Y. J. Lin, H. Y. Huang, T. H. Hsieh, Y. R. Liu, C. W. Liang, T. W. W. Chen, S. Yip, A. Lum, K. T. Kuo, Y. M. Jeng, S. C. Yu, Y. C. Chung, J. C. Lee, Clinicopathologic characterization of GREB1-rearranged uterine sarcomas with variable sex-cord differentiation. *Am. J. Surg. Pathol.* **43**, 928–942 (2019).

22. E. A. Goebel, S. Hernandez Bonilla, F. Dong, B. C. Dickson, L. N. Hoang, D. Hardisson, M. D. Lacambra, F. I. Lu, C. D. M. Fletcher, C. P. Crum, C. R. Antonescu, M. R. Nucci, D. L. Kolin, Uterine tumor resembling ovarian sex cord tumor (UTROSCT): A morphologic and molecular study of 26 cases confirms recurrent NCOA1-3 rearrangement. *Am. J. Surg. Pathol.* **44**, 30–42 (2020).

23. H. L. Sweeney, E. L. F. Holzbaur, Motor proteins. *Cold Spring Harb. Perspect. Biol.* **10**, a021931 (2018).

24. C. G. Almeida, A. Yamada, D. Tenza, D. Louvard, G. Raposo, E. Coudrier, Myosin 1b promotes the formation of post-Golgi carriers by regulating actin assembly and membrane remodelling at the *trans*-Golgi network. *Nat. Cell Biol.* **13**, 779–789 (2011).

25. C. Delestre-Delacour, O. Carmon, F. Laguerre, C. Estay-Ahumada, M. Courel, S. Elias, L. Jeandel, M. V. Rayo, J. R. Peinado, L. Sengmanivong, S. Gasman, E. Coudrier, Y. Anouar, M. Montero-Hadjadje, Myosin 1b and F-actin are involved in the control of secretory granule biogenesis. *Sci. Rep.* **7**, 5172 (2017).

26. L. Salas-Cortes, F. Ye, D. Tenza, C. Wilhelm, A. Theos, D. Louvard, G. Raposo, E. Coudrier, Myosin Ib modulates the morphology and the protein transport within multi-vesicular sorting endosomes. *J. Cell Sci.* **118**, 4823–4832 (2005).

27. K. A. Makowska, R. E. Hughes, K. J. White, C. M. Wells, M. Peckham, Specific myosins control actin organization, cell morphology, and migration in prostate cancer cells. *Cell Rep.* **13**, 2118–2125 (2015).

28. H. R. Zhang, S. Y. Lai, L. J. Huang, Z. F. Zhang, J. Liu, S. R. Zheng, K. Ding, X. Bai, J. Y. Zhou, Myosin 1b promotes cell proliferation, migration, and invasion in cervical cancer. *Gynecol. Oncol.* **149**, 188–197 (2018).

29. G. Ohmura, T. Tsujikawa, T. Yaguchi, N. Kawamura, S. Mikami, J. Sugiyama, K. Nakamura, A. Kobayashi, T. Iwata, H. Nakano, T. Shimada, Y. Hisa, Y. Kawakami, Aberrant myosin 1b expression promotes cell migration and lymph node metastasis of HNSCC. *Mol. Cancer Res.* **13**, 721–731 (2015).

30. X. Zhou, R. Wang, X. Li, L. Yu, D. Hua, C. Sun, C. Shi, W. Luo, C. Rao, Z. Jiang, Y. Feng, Q. Wang, S. Yu, Splicing factor SRSF1 promotes gliomagenesis via oncogenic splice-switching of MYO1B. *J. Clin. Invest.* **129**, 676–693 (2019).

31. J. B. Lubetsky, M. Swope, C. Dealwis, P. Blake, E. Lolis, Pro-1 of macrophage migration inhibitory factor functions as a catalytic base in the phenylpyruvate tautomerase activity. *Biochemistry* **38**, 7346–7354 (1999).

32. T. Calandra, T. Roger, Macrophage migration inhibitory factor: A regulator of innate immunity. *Nat. Rev. Immunol.* **3**, 791–800 (2003).

33. E. Cavalli, R. Ciurleo, M. C. Petralia, P. Fagone, R. Bella, K. Mangano, F. Nicoletti, P. Bramanti, M. S. Basile, Emerging role of the macrophage migration inhibitory factor family of cytokines in neuroblastoma. Pathogenic effectors and novel therapeutic targets? *Molecules* **25**, 1194 (2020).

34. S. Stosic-Grujicic, I. Stojanovic, F. Nicoletti, MIF in autoimmunity and novel therapeutic approaches. *Autoimmun. Rev.* **8**, 244–249 (2009).

35. M. R. Guda, M. A. Rashid, S. Asuthkar, A. Jalasutram, J. L. Caniglia, A. J. Tsung, K. K. Velpula, Pleiotropic role of macrophage migration inhibitory factor in cancer. *Am. J. Cancer Res.* **9**, 2760–2773 (2019).

36. R. Kleemann, A. Hausser, G. Geiger, R. Mischke, A. Burger-Kentischer, O. Flieger, F. J. Johannes, T. Roger, T. Calandra, A. Kapurniotu, M. Grell, D. Finkelmeier, H. Brunner, J. Bernhagen, Intracellular action of the cytokine MIF to modulate AP-1 activity and the cell cycle through Jab1. *Nature* **408**, 211–216 (2000).

37. M. W. Zimmerman, Y. Liu, S. He, A. D. Durbin, B. J. Abraham, J. Easton, Y. Shao, B. Xu, S. Zhu, X. Zhang, Z. Li, N. Weichert-Leahey, R. A. Young, J. Zhang, A. T. Look, *MYC* drives a subset of high-risk pediatric neuroblastomas and is activated through mechanisms including enhancer hijacking and focal enhancer amplification. *Cancer Discov.* **8**, 320–335 (2018).

38. C. L. Buchheit, K. J. Weigel, Z. T. Schafer, Cancer cell survival during detachment from the ECM: Multiple barriers to tumour progression. *Nat. Rev. Cancer* **14**, 632–641 (2014).

39. E. Piskounova, M. Agathocleous, M. M. Murphy, Z. Hu, S. E. Huddlestun, Z. Zhao, A. M. Leitch, T. M. Johnson, R. J. De Berardinis, S. J. Morrison, Oxidative stress inhibits distant metastasis by human melanoma cells. *Nature* **527**, 186–191 (2015).

40. H.-F. Zhang, C. S. Hughes, W. Li, J.-Z. He, D. Surdez, A. M. El-Naggar, H. Cheng, A. Prudova, A. Delaidelli, G. L. Negri, X. Li, M. S. Ørum-Madsen, M. M. Lizardo, H. Z. Oo, S. Colborne, T. Shyp, R. Scopim-Ribeiro, C. A. Hammond, A.-C. Dhez, S. Langman, J. K. M. Lim, S. H. Y. Kung, A. Li, A. Steino, M. Daugaard, S. J. Parker, R. I. K. Geltink, R. J. Orentas, L.-Y. Xu, G. B. Morin, O. Delattre, D.

S Dimitrov, P. H. Sorensen, Proteomic screens for suppressors of anoikis identify IL1RAP as a promising surface target in Ewing sarcoma. *Cancer Discov.* **11**, 2884–2903 (2021).

41. R. Zeid, M. A. Lawlor, E. Poon, J. M. Reyes, M. Fulciniti, M. A. Lopez, T. G. Scott, B. Nabet, M. A. Erb, G. E. Winter, Z. Jacobson, D. R. Polaski, K. L. Karlin, R. A. Hirsch, N. P. Munshi, T. F. Westbrook, L. Chesler, C. Y. Lin, J. E. Bradner, Enhancer invasion shapes MYCN-dependent transcriptional amplification in neuroblastoma. *Nat. Genet.* **50**, 515–523 (2018).

42. S. Masri, P. Sassone-Corsi, The emerging link between cancer, metabolism, and circadian rhythms. *Nat. Med.* **24**, 1795–1803 (2018).

43. G. Sulli, M. T. Y. Lam, S. Panda, Interplay between circadian clock and cancer: New frontiers for cancer treatment. *Trends Cancer* **5**, 475–494 (2019).

44. P. J. Skene, S. Henikoff, An efficient targeted nuclease strategy for high-resolution mapping of DNA binding sites. *eLife* **6**, e21856 (2017).

45. M.-T. Prospéri, P. Lépine, F. Dingli, P. Paul-Gilloteaux, R. Martin, D. Loew, H.-J. Knölker, E. Coudrier, Myosin 1b functions as an effector of EphB signaling to control cell repulsion. *J. Cell Biol.* **210**, 347–361 (2015).

46. J. Pernier, R. Kusters, H. Bousquet, T. Lagny, A. Morchain, J.-F. Joanny, P. Bassereau, E. Coudrier, Myosin 1b is an actin depolymerase. *Nat. Commun.* **10**, 5200 (2019).

47. J. Pernier, A. Morchain, V. Caorsi, A. Bertin, H. Bousquet, P. Bassereau, E. Coudrier, Myosin 1b flattens and prunes branched actin filaments. *J. Cell Sci.* **133**, jcs247403 (2020).

48. O. Iuliano, A. Yoshimura, M. T. Prospéri, R. Martin, H. J. Knölker, E. Coudrier, Myosin 1b promotes axon formation by regulating actin wave propagation and growth cone dynamics. *J. Cell Biol.* **217**, 2033–2046 (2018).

49. D. G. Stupack, T. Teitz, M. D. Potter, D. Mikolon, P. J. Houghton, V. J. Kidd, J. M. Lahti, D. A. Cheresh, Potentiation of neuroblastoma metastasis by loss of caspase-8. *Nature* **439**, 95–99 (2006).

50. K. C. Williams, M. A. Cepeda, S. Javed, K. Searle, K. M. Parkins, A. V. Makela, A. M. Hamilton, S. Soukhtehzari, Y. Kim, A. B. Tuck, J. A. Ronald, P. J. Foster, A. F. Chambers, H. S. Leong, Invadopodia are chemosensing protrusions that guide cancer cell extravasation to promote brain tropism in metastasis. *Oncogene* **38**, 3598–3615 (2019).

51. S. Seetharaman, S. Etienne-Manneville, Cytoskeletal crosstalk in cell migration. *Trends Cell Biol.* **30**, 720–735 (2020).

52. F. Weiss, D. Lauffenburger, P. Friedl, Towards targeting of shared mechanisms of cancer metastasis and therapy resistance. *Nat. Rev. Cancer* **22**, 157–173 (2022).

53. K. Eichelbaum, M. Winter, M. B. Diaz, S. Herzig, J. Krijgsveld, Selective enrichment of newly synthesized proteins for quantitative secretome analysis. *Nat. Biotechnol.* **30**, 984–990 (2012).

54. K. Eichelbaum, J. Krijgsveld, Rapid temporal dynamics of transcription, protein synthesis, and secretion during macrophage activation. *Mol. Cell. Proteomics* **13**, 792–810 (2014).

55. L. J. Steinbock, S. Krishnan, R. D. Bulushev, S. Borgeaud, M. Blokesch, L. Feletti, A. Radenovic, Probing the size of proteins with glass nanopores. *Nanoscale* **6**, 14380–14387 (2014).

56. F. J. Swartling, M. R. Grimmer, C. S. Hackett, P. A. Northcott, Q.-W. Fan, D. D. Goldenberg, J. Lau, S. Masic, K. Nguyen, S. Yakovenko, X.-N. Zhe, H. C. F. Gilmer, R. Collins, M. Nagaoka, J. J. Phillips, R. B. Jenkins, T. Tihan, S. R. Vandenberg, C. D. James, K. Tanaka, M. D. Taylor, W. A. Weiss, L. Chesler, Pleiotropic role for MYCN in medulloblastoma. *Genes Dev.* **24**, 1059–1072 (2010).

57. M. D. Taylor, P. A. Northcott, A. Korshunov, M. Remke, Y.-J. Cho, S. C. Clifford, C. G. Eberhart, D. W. Parsons, S. Rutkowski, A. Gajjar, D. W. Ellison, P. Lichter, R. J. Gilbertson, S. L. Pomeroy, M. Kool, S. M. Pfister, Molecular subgroups of medulloblastoma: The current consensus. *Acta Neuropathol.* **123**, 465–472 (2012).

58. P. A. Northcott, A. Korshunov, S. M. Pfister, M. D. Taylor, The clinical implications of medulloblastoma subgroups.*Nat. Rev. Neurol.* **8**, 340–351 (2012).

59. A. J. Camden, M. M. Szwarc, S. B. Chadchan, F. J. De Mayo, B. W. O'Malley, J. P. Lydon, R. Kommagani, Growth regulation by estrogen in breast cancer 1 (GREB1) is a novel progesterone-responsive gene required for human endometrial stromal decidualization. *Mol. Hum. Reprod.* **23**, 646–653 (2017).

60. M. J. Greenberg, G. Arpağ, E. Tüzel, E. M. Ostap, A perspective on the role of myosins as mechanosensors. *Biophys. J.* **110**, 2568–2576 (2016).

61. R. N. Kaplan, R. D. Riba, S. Zacharoulis, A. H. Bramley, L. Vincent, C. Costa, D. D. Mac Donald, D. K. Jin, K. Shido, S. A. Kerns, Z. Zhu, D. Hicklin, Y. Wu, J. L. Port, N. Altorki, E. R. Port, D. Ruggero, S. V. Shmelkov, K. K. Jensen, S. Rafii, D. Lyden, VEGFR1-positive haematopoietic bone marrow progenitors initiate the pre-metastatic niche. *Nature* **438**, 820–827 (2005).

62. Y. Liu, X. Cao, Characteristics and significance of the pre-metastatic niche. *Cancer Cell* **30**, 668–681 (2016).

63. H. M. Chan, J. Fan, Y. Xie, Y. X. Chen, W. Li, G. P. Jiang, Q. Liu, A. Meinhardt, P. K. H. Tam, Inhibition of tumor growth and metastasis in vitro and in vivo by targeting macrophage migration inhibitory factor in human neuroblastoma. *Oncogene* **25**, 3501–3508 (2006).

64. A. Delaidelli, G. L. Negri, A. Jan, B. Jansonius, A. El-Naggar, J. K. M. Lim, D. Khan, H. Z. Oo, C. J. Carnie, M. Remke, J. M. Maris, G. Leprivier, P. H. Sorensen, MYCN amplified neuroblastoma requires the mRNA translation regulator eEF2 kinase to adapt to nutrient deprivation. *Cell Death Differ.* **24**, 1564–1576 (2017).

65. S. Zhu, X. Zhang, N. Weichert-Leahey, Z. Dong, C. Zhang, G. Lopez, T. Tao, S. He, A. C. Wood, D. Oldridge, C. Y. Ung, J. H. van Ree, A. Khan, B. M. Salazar, E. L. da Rocha, M. W. Zimmerman, F. Guo, H. Cao, X. Hou, S. J. Weroha, A. R. Perez-Atayde, D. S. Neuberg, A. Meves, M. A. McNiven, J. M. van Deursen, H. Li, J. M. Maris, A. T. Look, LMO1 synergizes with MYCN to promote neuroblastoma initiation and metastasis. *Cancer Cell* **32**, 310–323.e5 (2017).

66. R. Patro, G. Duggal, M. I. Love, R. A. Irizarry, C. Kingsford, Salmon provides fast and bias-aware quantification of transcript expression. *Nat. Methods* **14**, 417–419 (2017).

67. C. Soneson, M. I. Love, M. D. Robinson, Differential analyses for RNA-seq: Transcript-level estimates improve gene-level inferences. *F1000Res.* **4**, 1521 (2015).

68. M. I. Love, W. Huber, S. Anders, Moderated estimation of fold change and dispersion for RNA-seq data with DESeq2. *Genome Biol.* **15**, 550 (2014).

69. P. Depuydt, J. Koster, V. Boeva, T. D. Hocking, F. Speleman, G. Schleiermacher, K. De Preter, Meta-mining of copy number profiles of high-risk neuroblastoma tumors. *Sci. Data* **5**, 180240 (2018).

70. C. S. Hughes, S. Foehr, D. A. Garfield, E. E. Furlong, L. M. Steinmetz, J. Krijgsveld, Ultrasensitive proteome analysis using paramagnetic bead technology. *Mol. Syst. Biol.* **10**, 757 (2014).

71. C. S. Hughes, S. Moggridge, T. Müller, P. H. Sorensen, G. B. Morin, J. Krijgsveld, Single-pot, solid-phase-enhanced sample preparation for proteomics experiments. *Nat. Protoc.* **14**, 68–85 (2019).

72. T. Suomi, G. L. Corthals, O. S. Nevalainen, L. L. Elo, Using peptide-level proteomics data for detecting differentially expressed proteins. *J. Proteome Res.* **14**, 4564–4570 (2015).
